# Genetic Data Reveal Nonlocal Juvenile Recruitment and Variable Seasonal Movement of a Highly Mobile Marine Fish Across Alaska

**DOI:** 10.1111/eva.70174

**Published:** 2026-01-06

**Authors:** Sara M. Schaal, Wes Larson, Johanna Vollenweider, Katharine Miller, Thilo Klenz, Jacek Maselko, Darcie Neff, Claire Tobin, Susanne McDermott, Ingrid Spies

**Affiliations:** ^1^ National Oceanographic and Atmospheric Administration National Marine Fisheries Service, Alaska Fisheries Science Center Seattle Washington USA; ^2^ Department of Anthropology University of Oklahoma Norman Oklahoma USA; ^3^ National Oceanographic and Atmospheric Administration National Marine Fisheries Service, Alaska Fisheries Science Center Juneau Alaska USA; ^4^ College of Fisheries and Ocean Sciences University of Alaska Fairbanks Fairbanks Alaska USA; ^5^ Alaska BioMap, Inc. Juneau Alaska USA; ^6^ School of Biological Sciences University of Utah Salt Lake City Utah USA

**Keywords:** amplicon panel, juvenile settlement, larval advection, Pacific cod, population genetics, seasonal migration

## Abstract

Movement patterns of marine fish are often difficult to accurately define given seasonal variation, ontogenetic shifts, and changing environmental conditions. However, outlining movement is crucial for understanding population dynamics, as well as for conservation and management efforts. Here, we evaluate seasonal adult movement and juvenile spatial distribution of Pacific cod ( *Gadus macrocephalus* ), a highly mobile and commercially important species, by developing and applying a genotyping‐in‐thousands by sequencing (GT‐seq) panel. This panel identifies four genetically distinct stocks within Alaska waters with high confidence in assignment (97% average accuracy across stocks). The application of this panel to adult, summer‐caught Pacific cod identified limited seasonal movement within and between populations, with the exception of those in the Northern Bering Sea (NBS). Two stocks occupied this region during the summer, non‐spawning season, and mixed at variable proportions in a west‐to‐east gradient potentially tied to the directionality of sea‐ice retreat in the NBS. Juvenile results indicated that although a predominant westward advection of larvae was prevalent in the Gulf of Alaska (GOA), two major deviations from this overall trend were apparent: (i) an eastward advection of a western GOA stock into the eastern GOA that varied interannually and (ii) a consistently high proportion of eastern GOA individuals in a western GOA narrow strait. These two deviating patterns suggest that mesoscale oceanographic processes play an important role in transport dynamics in the GOA that may be contrary to patterns expected based on the prevailing current. Taken together, our study provides novel insights into the movement dynamics of Pacific cod that can be leveraged by managers to help guide decision‐making for the species. Additionally, this inexpensive genetic panel can continue to be applied to further explore important questions about the ecology of Pacific cod in Alaska waters.

## 1 Introduction

Movement is an innate characteristic of marine fishes, but the extent and predictability of that movement can complicate efforts to understand and manage species. Movement throughout the lifespan of a marine fish is influenced by both passive and active mechanisms, with the relative role of these mechanisms often changing with ontogeny. Larval marine fish spend a period of their development in the water column, where they are subject to oceanographic dynamics (Gillanders et al. 2003). During this time, larval fish can exhibit ontogenetic diel vertical migrations (i.e., movements between surface and deeper waters that vary between life stages) that influence the extent of both their along‐shore and cross‐shore transport, which in turn can cause higher levels of local retention (i.e., juvenile settlement in the same region as their spawning site; Carr et al. 2008; Duffy‐Anderson et al. 2016; Huebert et al. 2011). Conversely, adult marine fish undergo mostly active transport through migrations in response to a number of cues, including responses to environmental changes (Perry et al. 2005; Tamario et al. 2019), in order to reach specific spawning habitats (Carpi et al. 2021; Lowerre‐Barbieri et al. 2021), and to find productive feeding grounds (Broms et al. 2012; Godø et al. 2004; Shimada and Kimura 1994). Taken together, movement in marine fishes is complex, and having a clear understanding of this movement and how it influences habitat use is challenging.

Delineating management boundaries is an integral and crucial part of managing marine fishes, but setting accurate boundaries is complicated when movement patterns for a given species are not well understood (Goethel et al. 2019). If stocks are not accurately defined and/or movement rates among stocks are poorly understood, assessment models used in management may not accurately reflect population dynamics (Goethel et al. 2011; Spies and Punt 2015; Spies et al. 2015). Identifying these patterns is especially important for species that are of both major economic and ecological significance, as is the case for Pacific cod ( *Gadus macrocephalus* ) in Alaska waters. Pacific cod is a key predator that is targeted in a highly valued US fishery, with the 2022 harvest totaling 403 million pounds valued at $225 million USD (Abelman et al. 2023). The species is currently managed as three separate stocks in Alaska waters—the Gulf of Alaska (GOA), Bering Sea, and Aleutian Islands—but initial tagging data suggest that individuals traverse across these management boundaries (Shimada and Kimura 1994). Although these data give initial insight into movement patterns, a comprehensive understanding of the extent, seasonality, and predictability of this movement across these stock boundaries for Pacific cod is still lacking.

Mechanisms influencing movement patterns in Pacific cod vary with ontogeny; some life stages contribute to limited movement and others to long‐distance transport. The spawning life history of Pacific cod promotes local retention due to their demersal spawning, where females deposit a single batch of slightly adhesive, negatively buoyant eggs that develop on the benthos and hatch in 3–4 weeks (Laurel et al. 2008). Conversely, the subsequent larval stage results in higher levels of transport due to newly hatched larvae migrating to the upper water column in as early as a single day post‐hatch (Hurst et al. 2010), with a pelagic larval duration of approximately 70 days (Laurel et al. 2008). During this time, both active and passive movements occur with larval fish exhibiting diel vertical migrations (Hurst et al. 2010) and transport via currents to coastal nursery grounds (Hinckley et al. 2019). The prevailing currents in the GOA suggest that larval fish primarily experience westward movement via the Alaska Current and the Alaska Coastal Current (ACC) (Ladd et al. 2005). Oceanographic models for Pacific cod larval dispersal predict mostly a pattern of local retention to adjacent nearshore nursery areas with some westward spillover (Hinckley et al. 2019). However, there has been little direct empirical evidence put forward to support results from these models.

Movement patterns of Pacific cod have also been inferred from population genetic data, which has identified an overall pattern of isolation by distance punctuated by genetic breaks in some areas (Cunningham et al. 2009; Drinan et al. 2018; Spies 2012; Spies et al. 2021). This suggests a limited amount of gene flow between neighboring populations. Mechanisms underlying limited gene flow can be caused by a number of factors, which cannot be informed by genetic analysis of adult spawners alone. First, local retention can occur where juveniles are self‐recruiting to their natal populations, and as adults, they remain in that region for spawning (Warner and Cowen 2002). Second, juveniles may settle in heterospecific regions (i.e., regions occupied by other populations) due to oceanographic transport of larvae, but differential survival after settlement causes local genotypes to survive. Finally, adults may migrate freely outside of the spawning season, but return to the same site (i.e., spawning‐site fidelity) or to their natal spawning site (i.e., natal homing) for spawning each year. To disentangle these mechanisms and identify their relative role in shaping population genetic patterns, additional data are needed.

Current evidence to inform these mechanisms has focused on adult movement dynamics inferred through tagging data. Mark‐recapture studies have found an overall pattern of intra‐basin (e.g., within the Eastern Bering Sea (EBS)) annual migrations from winter/spring spawning grounds to summer feeding grounds, but a small subset of tagged individuals migrated between basins (Rand et al. 2014; Shimada and Kimura 1994). This movement pattern suggests the potential for a partial migration strategy (i.e., individual variation in the extent of seasonal migration). Recent satellite tagging efforts have provided additional evidence that migrations between the GOA and the Bering Sea may be more common (S. McDermott & J. Nielsen *pers. comm*.). Although these data provide initial evidence for between‐basin movement, these studies are based on a limited number of samples due to the cost of both the tags themselves and their deployment/recapture.

Genetic stock identification (GSI) is a complementary method to tagging that can provide additional evidence for seasonal movement patterns and does so on a larger scale. While whole‐genome sequencing can be prohibitively costly for large‐scale studies on hundreds to thousands of individuals, targeted sequencing methods (e.g., amplicon sequencing) provide a powerful and cost‐effective alternative by scaling down the number of genetic markers and scaling up the number of samples for efficiently answering many research questions. Targeted sequencing methods require knowledge of the population genetic structure of the species in order to design a panel of genetic markers that can effectively differentiate the genetic stocks (i.e., unique spawning groups identified based on genetic differentiation) present in the region(s) of interest (Anderson et al. 2025; Bootsma et al. 2020). Marker panels that accurately delineate genetic stocks can be applied to samples collected at different life stages, locations, and seasons to gain a clearer understanding of movement patterns across space, time, and ontogeny. Therefore, combining genetic and tagging data is a powerful way to help disentangle mechanisms driving population genetic structure and identify appropriate stock boundaries. This combinatorial approach was used to provide strong evidence for natal homing in a closely related species, Atlantic cod ( *Gadus morhua* ), and provide managers with a clearer picture of habitat use in that system (Bonanomi et al. 2016).

In order to provide a more comprehensive understanding of Pacific cod movement dynamics, our study designs and implements a targeted sequencing panel to evaluate movement at multiple life stages. To do this, we first evaluated the population genomic structure of Pacific cod caught on spawning grounds using low‐coverage whole‐genome sequencing (lcWGS). These data were then used to build a Genotyping‐in‐Thousands by sequencing panel (GT‐seq panel) for Pacific cod that can be applied to this and future studies as a cost‐effective genetic resource for delineating the genetic stock of origin of thousands of individuals. Finally, using the newly designed GT‐seq panel, our study assessed two major research questions: (i) what is the seasonal variation in stock‐specific adult habitat use?, and (ii) what is the stock‐specific habitat use of juvenile Pacific cod throughout the GOA?

## 2 Methods

### 2.1 Sampling

**FIGURE 1:**
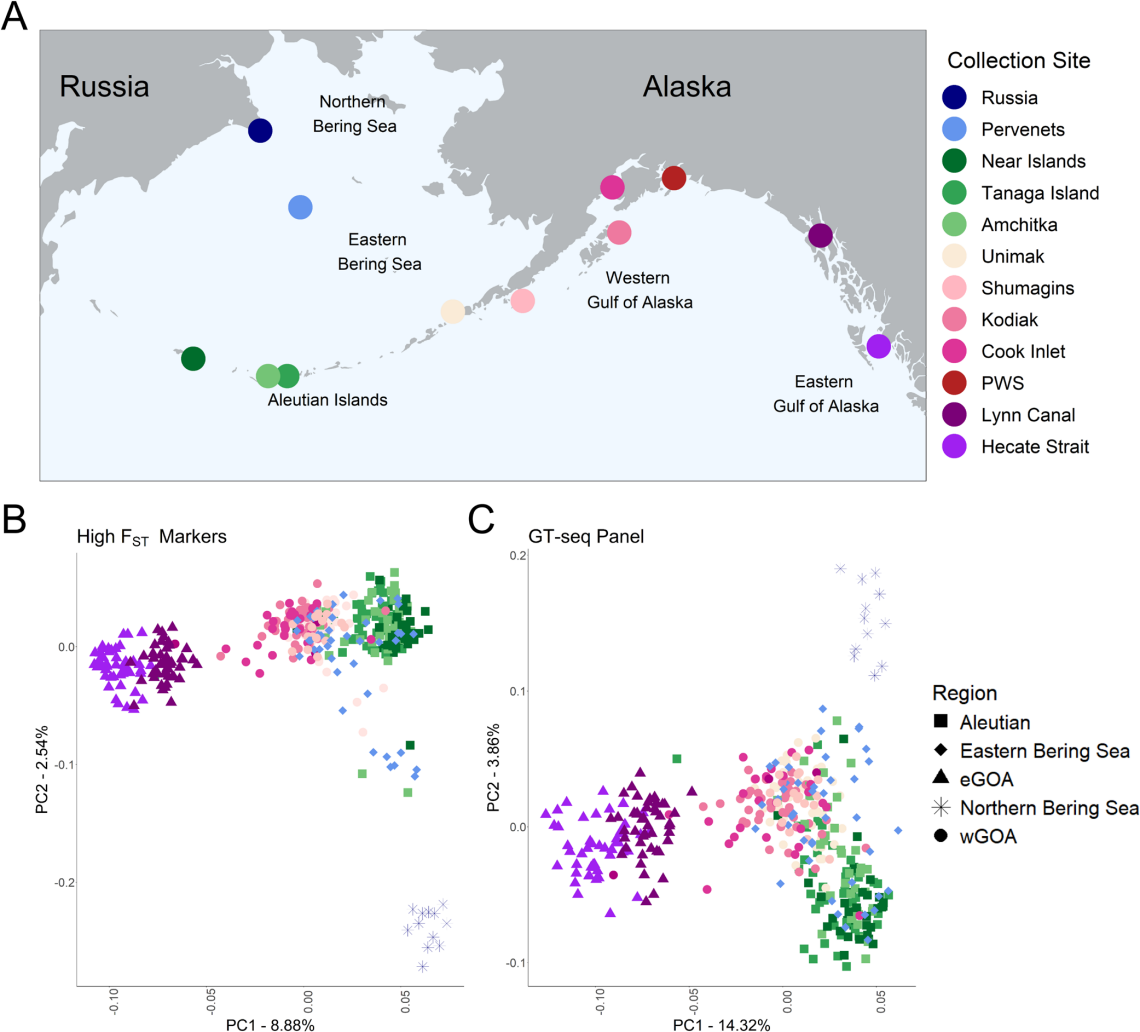
Visualization of GT‐seq panel population structure in comparison with baseline population low‐coverage whole‐genome sequencing data. (A) Map of sampled populations for GT‐seq panel development, (B) principal component analysis of top 0.2% high *F*_ST_ markers, 9519 SNPs, which outlines the target reporting groups for GT‐seq panel development, and (C) final GT‐seq panel after primer design and optimization.

Samples that were sequenced for lcWGS were obtained from fin clips or muscle plugs from 398 individuals spanning 12 sampling locations in the Bering Sea and GOA (Figure 1A; Table 1 “N (lcWGS)”). All samples were collected either by the National Oceanic and Atmospheric Administration (NOAA) winter trawl surveys or from fishery partners from the Freezer Longline Coalition (Table 1 “Collection Method”) during the winter spawning season between January and April (Table 1 “Sample Date”). Hereafter, this dataset is referred to as the panel design dataset.

**TABLE 1:** List of sample locations and sizes for spawning adults used to design the GT‐seq panel. *N* (lcWGS) = number of samples used in panel design with lcWGS, and *N* baseline = number of samples for the assignment baseline dataset for GT‐seq. Latitude and longitude are in degrees decimal.

Sample region	Sample site	*N* (lcWGS)	*N* (GT‐seq)	Latitude	Longitude	Sample date	Collection method
Northern Bering Sea	Russia	12	13	62.36	179.54	Jan‐18	Fishery
	St. Lawrence—Tagged Fish	0	15	63.75	−170.32	Jun‐19	Survey
Eastern Bering Sea	Pervenets Canyon	42	83	59.35	−175.31	Mar‐16	Fishery
	Zhemchug	0	59	57.66	−175.31	May‐17	Fishery
	Pribilof	0	53	57.04	−169.86	Apr‐17	Survey
Aleutian Islands	Near Islands	33	37	52.53	174.15	Feb‐05	Fishery
	Tanaga Island	41	104	51.67	−178.27	Feb‐05	Fishery
	Amchitka Pass	41	41	51.67	−179.82	Feb‐05	Fishery
	Adak	0	44	52.1	−175.86	Mar‐23	Fishery
Western Gulf of Alaska	Unimak	44	48	54.78	−164.91	Feb‐18	Fishery
	Shumagins	18	22	55.28	−159.28	Mar‐19	Fishery
	West Kodiak	0	49	57.2	−152.58	Feb‐22	Survey
	Kodiak	46	50	58.3	−151.5	Feb‐20	Fishery
	Cook Inlet	25	47	60.16	−152.05	Feb‐20	Fishery
	Prince William Sound	3	0	60.53	−147.08	Feb‐20	Fishery
Eastern Gulf of Alaska	Lynn Canal	46	47	58.17	−135.27	Feb‐20	Fishery
	Hecate Strait	47	75	53.13	−130.57	Mar‐04	Survey

We then genotyped the same samples analyzed with lcWGS as well as three sets of additional samples with GT‐seq: (1) additional samples taken during the spawning season, (2) adult samples collected outside of the spawning season, and (3) juvenile samples collected outside the spawning season. The additional spawning season samples consisted of 389 individuals collected from the NOAA winter trawl surveys or from fishery partners from locations within our reference sampling region (Table 1; Figure S1). The second dataset consisted of 470 adult samples caught in the summer non‐spawning season from the Bering Sea and GOA (Table 2). Fin clips or muscle plugs were collected from all individuals and were caught either via the NOAA Fisheries Alaska Fisheries Science Center Summer Bottom Trawl Surveys in 2021 and 2022 or from the International Pacific Halibut Commission longline survey in 2016 and 2018. The third dataset was used to determine the stock‐specific habitat use of juvenile Pacific cod and consisted of 657 fin clips from samples collected in juvenile beach seine surveys in the summers of 2021 and 2023 in the GOA (Table 3).

**TABLE 2:** List of sample locations, dates, and sizes for mixture collections of summer‐caught adult Pacific cod.

Sample site	*N*	Collection year	Latitude	Longitude
1. Adak	96	2018	51.89	−176.85
2. West Attu	47	2016	53.01	−172.32
3. St. Lawrence	27	2021	63.91	−165.49
4. South Central NBS	12	2021	60.99	−169.43
5. West of Nome	59	2021	64.41	−166.84
6. East of St. Lawrence	25	2021	62.99	−166.51
7. Norton Sound	30	2021	63.92	−165.5
8. Eastern Bering Sea	25	2021	56.68	−160.98
9. Western GOA	24	2021	54.82	−161.41
10. Kodiak	23	2021	57.37	−152.24
11. Outside Cook Inlet	23	2021	59.12	−151.86
12. Central GOA	54	2021	59.14	−149.24
13. Eastern GOA	25	2021	57.48	−136.88

**TABLE 3:** List of sample locations and sizes for mixture collections of juvenile Pacific cod.

Sample site	*N*	Latitude	Longitude
1. Near Sand Point 2021	69	55.1872	−159.6388
2. Mitrofania Bay 2021	22	55.9045	−158.7845
3. Sutwik Island 2021	47	56.58	−157.24
4. South of Kodiak 2021	35	57.11	−156.4591
5. Shelikov Strait 2021	51	58.0649	−154.5924
6. Shelikov Strait 2023	57	58.2586	−152.8526
7. North Kodiak 2021	57	58.2161	−152.4663
8. Cook Inlet outside 2021	125	59.3504	−150.7775
9. PWS outside 2021	51	60.0798	−148.0422
10. eGOA outside 2023	97	57.7943	−136.2386
11. eGOA outside 2021	29	57.4024	−135.8203
12. eGOA inside 2023	17	57.29	−133.82

### 2.2 Whole‐Genome Analysis and Panel Marker Selection

For the panel design dataset, DNA was extracted using a Qiagen DNeasy 96 Blood and Tissue Kit (Qiagen Inc., Valencia, CA). DNA was quantified using PicoGreen fluorescence on a BioTek Synergy HTX microplate reader (Agilent, Santa Clara, CA) and quality was assessed using gel electrophoresis on a 2% agarose gel. For each sample, DNA quantity was normalized to 10 ng based on microplate reader quantifications and then used to build libraries for lcWGS. Whole‐genome resequencing library preparation was conducted using methods similar to Therkildsen and Palumbi (2017), and Euclide et al. (2023). First, tagmentation was used to fragment the DNA with an Illumina Tagmentation Enzyme TDE1 (Illumina Inc., San Diego, CA) and tag the fragments with a universal overhang (partial adapter) complementary to the index primer sequences. Dual‐index primers were then added using PCR, followed by a second reconditioning PCR to minimize sequencing artifacts and add P5 and P7 primer sequences (Nextera, Illumina Inc., San Diego, CA). Next, the PCR products were normalized using SequalPrep Normalization Plate Kit (Applied Biosystems, Waltham, MA), pooled, and then cleaned using 0.6X AMPure XP beads (Beckman Coulter, Indianapolis, IN). The final cleaned library was visualized on an E‐Gel Precast Agarose Gel Electrophoresis System (Invitrogen, Waltham, MA) and quantified on a Qubit 2.0 (Invitrogen, Waltham, MA) using the dsDNA HS Quantification Assay Kit (Invitrogen, Waltham, MA). The final library was sequenced on a lane of Novaseq 6000 using 2 × 150 bp paired‐end sequencing (Illumina Inc., San Diego, CA).

Processing and filtering of lcWGS data were conducted using methods similar to Timm et al. (2024). For all samples, raw sequencing qualities were assessed with *fastqc* (v. 0.11.9) before and after adapter trimming with *trimmomatic* (v. 0.39). Trimmed sequences were aligned to the Atlantic cod genome (GadMor3.0 GenBank assembly: GCA_902167405.1) with *bwa‐mem* (*bwa* v. 0.7.17). Unmapped reads were filtered using *samtools view* (v. 1.11), and duplicate reads were removed using *picard MarkDuplicates* (v. 2.23.9). Aligned reads were then used to calculate genotype‐likelihoods and identify single‐nucleotide polymorphisms (SNPs) using *ANGSD* (v. 0.933) with the *samtools* genotype likelihood model (−GL 1). Loci were filtered based on a minor allele frequency > 0.05 (‐Maf), minimum depth to the number of individuals (*N* = 398, ‐setminDepth), maximum depth to the number of individuals times 20 (*N* = 398*20, ‐setmaxDepth), mapping quality of > 15 (‐minMapQ), and a base quality score of > 15 (‐minQ). Finally, SNPs were called if the likelihood ratio test had a *p*‐value < 1e^−10^.

The resulting polymorphic SNPs were used to assess the population genomic structure of Pacific cod across our collection sites. First, we used all SNPs in a principal component analysis (PCA) using *pcangsd* (v. 0.99). Next, we assessed levels of admixture between individuals among collections using *NGSadmix* (v. 32) varying K from 2 to 5. Finally, we performed by‐site *F*_ST_ calculations in *ANGSD* (v. 0.933) for all pairwise comparisons in our dataset to calculate a metric that could be used to choose loci for inclusion in a GT‐seq panel based on their discriminatory power. This was done by first estimating the site allele frequency (SAF) likelihood for each sampling location using the *realSFS* function (‐doSaf 1). We then used the per‐location SAF as input to calculate the two‐dimensional site frequency spectrum (‐realSFS) for each pairwise comparison in the dataset. Finally, we calculate by‐site *F*_ST_ using the same thresholds that were used in the genotype likelihood calculations using the *realSFS fst* function.

We used data from the SNPs identified from lcWGS data to develop a GT‐seq panel that contained 200–300 SNPs and that could recapitulate the patterns of population structure found in the full lcWGS dataset. This number of loci was chosen because it represents a reasonable number of primers to multiplex based on previous experience and would enable efficient sequencing with adequate coverage for calling genotypes. Loci for the GT‐seq panel were identified using per‐locus *F*_ST_ values to maximize resolution for differentiating populations. Per locus *F*_ST_ was calculated using *ANGSD* (v. 0.933) with the *realSFS* function across all pairwise comparisons (i.e., for each locus, 55 different *F*_ST_ values were calculated). We downsampled the loci with the following steps to identify loci for the panel: (1) identified the 10 loci with the highest *F*_ST_ per chromosome per pairwise comparison, 10 loci × 23 chromosomes × 55 comparisons = 12,620 loci, (2) subset based on unique loci resulting in 7872 loci, (3) removed loci with *F*_ST_ < 0.2 because some pairwise comparisons have very low overall *F*_ST_ resulting in removing 3148 loci, and (4) each chromosome was then split into six equal windows, and the two loci with the highest *F*_ST_ values in each window were retained (i.e., 6 windows × 2 loci × 23 chromosomes = 276 loci) where only one locus can come from a given pairwise comparison for each chromosome. The latter step prevents a single pairwise comparison from over‐contributing to the final set of loci per chromosome. We chose this approach rather than an approach focused on identifying only loci differentiating major stock groups to reduce ascertainment bias and ensure that our GT‐seq panel reflected overall patterns of population structure rather than only structure among major stocks.

After choosing the 276 loci, we performed a PCA on those loci and compared that to a PCA based on a larger subset of high‐resolution SNPs (9519 loci) to ensure that the marker panel accurately captured the population genomic structure present in the genome. This subset was composed of the SNPs that were in the top 0.2% of the *F*_ST_ distribution for at least one of the 55 pairwise comparisons to ensure that SNPs were not preferentially chosen from comparisons with higher differentiation. We then assessed the genome for areas of especially high differentiation that were noted in previous studies (Spies et al. 2021) to ensure that we captured divergence between certain geographic locations that was concentrated within tight genetic regions as opposed to genome‐wide divergence. This was to ensure the panel could resolve all the genomic structures present in the Pacific cod genome. Finally, the zona‐pellucida sperm‐binding protein 3 gene (ZP3) was assessed for outliers, and any outlier loci from that region were added to the panel because this region has been shown in previous studies to capture latitudinal structure in Pacific cod (Spies et al. 2021). Ten additional loci were added to the panel from these assessments resulting in a panel of 286 loci.

### 2.3 Primer Design and Panel Optimization

Using this initial set of 286 loci, primers were designed using Geneious prime (v. 2022.1.1), with a target product size of 110–120 bp, a minimum primer size of 18 bp, a maximum primer size of 25 bp, a minimum, maximum, and optimal *T*_m_ of 57°C, 63°C, and 60°C, respectively, and a minimum, maximum, and optimal GC content of 20%, 80%, and 50%, respectively. If a primer option fit these criteria, but had any off‐target binding, it was removed from further consideration. If a given locus did not have a primer set that fit within these requirements, the locus was dropped from the panel.

Primer combinations that passed our criteria were ordered at a concentration of 100 μM from Integrated DNA Technologies (IDT), aliquoted and diluted to a concentration of 200 μM, and finally mixed in equal proportions and diluted to a stock concentration of 200 μM per primer. Optimization of the GT‐seq panel was carried out with 95 Pacific cod samples that included samples from two genetic groups and a negative control. All samples were extracted using the Qiagen DNeasy 96 Blood and Tissue Kit (Qiagen Inc., Valencia, CA), and template DNA was then dried for input in the GT‐seq protocol. DNA was first amplified with GT‐seq primers pooled in equal proportions for 15 cycles of PCR. Then a second round of PCR added individual barcodes to the 96 samples using custom i5 and i7 indexed primers (Campbell et al. 2015). After both rounds of PCR, normalization was performed using the SequalPrep Normalization Plate Kit (Applied Biosystems, Waltham, MA). Normalized DNA was pooled in equal proportions, and a 0.5× bead clean up using AMPure Beads (Beckman Coulter, Indianapolis, IN) was used for size selection of our libraries to remove any small fragments of primer dimer. Final libraries were quantified on a Qubit 2.0 (Invitrogen, Waltham, MA) using the dsDNA HS Quantification Assay Kit (Invitrogen, Waltham, MA). Paired‐end 2 × 150 bp sequencing was performed on an Illumina MiSeq (Illumina Inc., San Diego, CA) with each sample grouped by index using the MiSeq Analysis Software (Illumina Inc., San Diego, CA).

Raw paired‐end fastq files were first merged using *flash* (v. 1.2.11; using the following non‐default flags *‐m* 10 *‐M* 100). Successfully paired‐end reads were then mapped using *bwa‐mem* (v. 0.7.17; using the following non‐default flags *‐a ‐M*) to a reference FASTA file that included, for each target SNP, 150 bp up and downstream of the SNP. The sam files created from the alignment were converted to bam files using *samtools view* (v. 1.11), sorted using *samtools sort*, and then indexed using *samtools index*. Primer pool optimization was performed until the panel contained no highly over‐amplifying primer combinations and no primer combinations that contained high numbers of off‐target hits. Any loci that were well outside the distribution of amplification (i.e., read counts) across loci were dropped due to over‐amplification. The amount of off‐target hits was assessed by comparing the number of raw reads to those that successfully merged after paired‐end alignment for each locus. Any loci that had inflated raw to merged read ratios suggest that both primers for that combination did not anneal in the proper location, resulting in off‐target hits. These primer combinations were therefore dropped from the panel.

### 2.4 Panel Performance

The panel was evaluated using 787 spawning samples from known locations, which included all of the panel design samples, additional samples from those locations that failed during whole‐genome sequencing, but were successfully sequenced with the GT‐seq panel, and five additional sampling sites (Table 1 “N (GT‐seq)”). The additional sampling sites included two new locations in the EBS, Zhemchug and Pribilof; one in the western GOA (wGOA), West Kodiak; and one in the Aleutian Islands, Adak (Table 1). Additionally, in order to increase the sample size of the Northern Bering Sea (NBS) sample set, summer non‐spawning individuals were added that showed genetic similarity to NBS samples in lcWGS data (Table 1; St. Lawrence—Tagged Fish). Together, these samples are hereafter referred to as the reference dataset. All reference samples were prepared following the GT‐seq protocol outlined above and sequenced using an Illumina MiSeq. The resulting bam files were used to call SNPs using *freebayes* (v. 1.3.6; using the following non‐default flags ‐‐haplotype‐length 0 ‐kwVa ‐X ‐u ‐i). Assessment of panel performance and all downstream analyses were performed in R (v. 4.2.1; R core development team 2016). Microhaplotypes were assembled and filtered using the R function *prepHaplot* from the program *microhaplot* (Ng et al. https://doi.org/10.5281/zenodo.820110) using the variant call format (VCF) file output. Microhaplotypes are multi‐allelic markers that combine nearby SNPs from short‐read sequencing data (Baetscher et al. 2018; Kidd et al. 2013). Therefore, although a primer combination is designed to target an individual SNP of interest, additional SNPs from the sequences around it can be leveraged to increase analytical power (Anderson et al. 2025; Baetscher et al. 2018; Kidd et al. 2013). Individual‐level data were first filtered to retain haplotypes with at least 20 reads at a locus. Second, the resulting haplotypes were filtered to remove any with a read depth ratio of less than 0.2 within an individual. This removes haplotypes that have a depth ratio less than 0.2 when compared to the read depth of the haplotype with the highest‐read depth (see Baetscher et al. 2018 for a detailed description). Genotypes were called from the remaining haplotypes as either heterozygous for the two highest‐read depth alleles or homozygous if the individual only had sequences for a single haplotype allele. Once all individuals were genotyped, individuals were retained only if they had successfully called genotypes at > 75% of loci. Next, loci were retained only if > 75% of remaining individuals were successfully genotyped. Finally, if any locus showed significant deviations from Hardy–Weinberg equilibrium (HWE) in greater than half of our total sampled reference collections (i.e., 8), it was removed (Larson et al. 2014).

All individuals and loci that passed these filters were then used in a PCA to determine whether the population genomic structure that the panel was designed for was still evident with the reduced number of markers and increased number of individuals. The clusters present in the whole‐genome PCA were used as reporting groups for initial tests of GSI. We then used an iterative approach to assess possible reporting groups. We initially attempted to construct reporting groups that included the EBS and wGOA as separate groups because differentiating these groups is of interest to management, and some individuals did form a unique cluster. However, it was clear that EBS and wGOA populations could not be accurately differentiated from initial stock identification evaluations (62.5% and 66.3% accurate assignment respectively). This was not surprising given the lack of discrete clusters for these sites in PCAs. We then combined these groups and tested four reporting groups that corresponded well to the population clusters found in PCAs: (1) NBS, (2) Aleutian Islands, (3) EBS/wGOA, and (4) eastern GOA (eGOA). To assess stock identification accuracy, we used a simulated mixture analysis with a leave‐one‐out, cross‐validation approach using the *assess_reference_loo* function in the program *Rubias* (v. 0.3.3; Anderson et al. 2008). This assessment method outputs an essentially unbiased estimate of the GSI accuracy through its use of cross‐validation compared to traditional GSI assignment‐based methods that use parametric bootstrap with baseline resampling (see detailed comparison in Anderson et al. 2008). In order to preserve missing data patterns in our reference dataset, we resampled based on “individuals” for the *resampling_unit* flag of the program. The simulated individual reporting units were compared to the known reporting units to determine the accuracy of GSI with the GT‐seq panel. The final reported accuracy is based on individuals who were assigned to a genetic reporting group with > 90% confidence. Lastly, we tested whether loci that were in linkage disequilibrium (LD) impacted the assignment accuracy of our panel. LD was calculated pairwise for each locus and all loci on the same chromosome using the *pair.ia()* function, which calculates the index of association from the *poppr* package (v. 2.9.6) in R. Loci that were significantly linked were dropped, and assignment accuracy was recalculated to determine the impact of linkage on panel performance.

### 2.5 Unknown Origin Sample Sequencing

The resulting GT‐seq panel was genotyped on the two datasets of individuals of unknown genetic stock using the methods outlined above. The reference VCF file created with the reference dataset was then used to assemble microhaplotypes to be used as genotypes for each marker region (i.e., the sequence amplified by each primer pair) for every individual. Once all individuals were genotyped, they were retained only if they had successfully called genotypes at > 75% loci. Next, loci were retained only if > 75% of remaining individuals were successfully genotyped. Mixture collections required a minimum of 10 individuals; therefore, after filtering for missing data, samples were binned based on geographic proximity until a sufficient sample size was reached. In order to assign individuals back to their spawning stock of origin, the microhaplotypes were used with the *infer_mixture()* function with default options from the program *Rubias* (Moran and Anderson 2019), which uses Markov Chain Monte Carlo (MCMC) estimation to output both the log‐probability of individual assignment to a genetic reporting group and the overall mixing proportions of each mixture collection. We used the log‐probability values to filter for only those individuals that had > 90% confidence in assignment. In addition, we assessed the *z*‐score (*z*‐statistic calculated from the expected log‐likelihood and its standard deviation) to filter any individuals that were assigned to a population that may not have been in our reference dataset (Moran and Anderson 2019). Therefore, individuals with *z*‐score values outside of an expected range of either > 5 or < −5 were also removed from downstream analysis.

### 2.6 Summer‐Caught Adult Analysis

For the summer‐caught adult samples, a Pearson's Chi‐squared test was used to assess whether seasonal movement was occurring from each mixture collection with the *chisq.test()* function. A success was counted when an individual's assigned genetic reporting group matched the region they were collected from, and a failure was counted when an individual was assigned to any other genetic reporting group that was not the region they were collected from. For example, in the summer collection from the eGOA, when an individual was assigned to the eGOA it was counted as a success, but if it was assigned to any other reporting group, it was assigned a failure. These values were compared to the winter (reference dataset) counterpart. The winter counterpart was determined by which general region the mixture collection came from. For example, there are five sample sites in the NBS from the summer mixture collections, and all of those sites were individually compared to the NBS winter (reference dataset) collection. If the test was significant (i.e., *P*‐value < 0.05), it indicated that the distribution of genetic stocks shifts seasonally in that mixture collection location. If the data for any given comparison violated the assumption of fewer than 5 expected values of the Pearson's Chi‐squared test, we used Monte Carlo simulations to simulate the distribution of expected *P*‐values for that comparison using the *simulate.p.value = TRUE* flag of the *chisq.test()* function, and we simulated 5000 replicates.

### 2.7 Juvenile Analysis

For juvenile samples, Lagrangian particle integrations were performed in order to assess whether oceanographic conditions explained patterns found in the proportion of reporting group assignments in each mixture collection. Hybrid Coordinate Ocean Model (HYCOM) three‐hourly horizontal velocity output (publicly available at https://www.hycom.org) was used to integrate particles using OpenDrift (Dagestad et al. 2018), an open‐source Python‐based numerical particle integrator. Particle integrations were started on the 15th of the months of January, February, March, and April for three years that span our sample collections, 2021–2023 (Table 3). A total of 17,000 particles were released at fixed depths between 0 to 40 m in a cluster centered on the northeast corner of Kodiak Island, 58.3 N 151.5 W. Forward integrations were started on the 15th of each month, and simulations were run for a total of 200 days, and no vertical movement was prescribed to the particles.

## 3 Results

### 3.1 Population Structure

lcWGS of 398 individuals spanning 12 geographic locations (Figure 1A) resulted in 1,923,025 SNPs and an average coverage of 2.3X per individual. Analysis of all loci in the reference dataset resulted in four distinct genetic stocks (Figure S2), which became even more distinct when evaluating high *F*_ST_ markers (Figure 1B): (1) NBS, (2) Aleutian Islands, (3) wGOA/EBS, and (4) eGOA. This was also supported by admixture analyses that delineated these four groups, but also showed evidence of some mixing of the NBS with Pervenets in the EBS (Figure S3). Genetic research has previously reported no genetic difference between NBS and EBS individuals when analyzing data using a discriminant analysis of principal components (DAPC) where sampling locations were designated as priors (Spies et al. 2020). However, upon reevaluation of these data using a PCA, we uncovered a similar pattern found in our data where an EBS sampled location (i.e., Pervenets) contained two genetically distinct groups: one more similar to a wGOA/EBS genotype and another to a NBS genotype (Figure S4). This is also evident when assessing admixture, where we have a subset of individuals from Pervenets that have a high proportion of genetic variance from the NBS genetic cluster in some individuals (see *K* = 5 red bars in the Pervenets samples; Figure S3). The four genetic stocks identified above were used to design the initial GT‐seq panel and assess panel performance. The initial set of sites used in the final GT‐seq panel also supported these groupings (Figure 1C).

### 3.2 Panel Design and Optimization

During primer design, 17 loci were excluded because no usable primer combinations were identified to amplify those target loci based on our criteria outlined in the Methods. Therefore, the initial panel consisted of 269 loci (5.9% primer dropout during design). Primer pool optimization was performed three times before the panel contained no over‐amplifying primer combinations and no primer combinations that contained off‐target hits. During these optimization steps, 26 primer combinations were dropped from the first optimization, an additional 16 from the second, and an additional 2 from the third. Therefore, the primer multiplex used for all downstream analyses consisted of 225 primer combinations (15.3% primer dropout during multiplex optimization). During the initial analysis of the reference dataset, an additional 26 loci dropped out either due to poor mapping, being monomorphic in the reference dataset, or not passing filtering criteria set in *freebayes* (see Methods for filters), resulting in a final panel of 199 markers (11.6% loci dropout during initial analysis).

An additional 28 markers were removed because they were not present in > 75% of individuals, and 8 loci were removed because they deviated significantly from HWE, resulting in a final panel of 178 markers containing 648 unique alleles. Microhaplotypes contained an average of 3.6 alleles (SD = ±5.98). For all markers, the overall *F*_ST_ of markers among collections was 0.81 (SD = ±0.092) and the H_o_ was 0.42 (SD = ±0.18).

### 3.3 Panel Performance

When assessing the lcWGS data for inclusion in the assignment reference dataset, there were 26 individuals that had intermediate genotypes between the NBS and the wGOA/EBS. These individuals were dropped from the downstream analyses. PCA and collection site pairwise *F*_ST_ values based on genotyping all reference individuals with GT‐seq loci revealed our four reporting groups were still the dominant signal in the dataset (Figures S5 and S6, respectively). Using the leave‐one‐out simulation approach for assignment, the panel performed with 95.6% accuracy across all individuals in the reference dataset for these four reporting groups. When filtered for individuals that had > 90% confidence in their GSI, the panel performed with 96.97% accuracy overall (Table 4; Figure S7). Individuals reported to the Aleutian Islands with 97.6% accuracy, to the eGOA with 98.9% accuracy, to the NBS with 97.1% accuracy, and to the wGOA/EBS group with 90.7% accuracy (Table 4; Figure S7). Attempts were made to add a fifth reporting group to our GT‐seq panel in order to split the EBS and wGOA because these are separate management units, but this significantly reduced assignment accuracy (63.5% and 66.3% assignment accuracy for EBS and wGOA, respectively). LD analyses revealed 43 loci that were significantly linked. However, assignment accuracies were not impacted by the presence of these loci (overall accuracy dropped to 96.81%). Therefore, we kept loci in LD in the panel.

**TABLE 4:** Assignment accuracies for each reporting group and overall (in bold) for samples that were assigned to their respective reporting group with > 90% confidence.

Reporting group	Assignment accuracy (%)
Aleutian Islands	97.6
Northern Bering Sea	97.1
Eastern Gulf of Alaska	98.9
Western Gulf of Alaska/Eastern Bering Sea	90.7
**Overall**	**97.0**

### 3.4 Summer‐Caught Adult Sample Assignment

Sixteen loci and six individuals were dropped from the analysis of the adult summer samples due to < 75% of individuals genotyped and < 75% of loci genotyped, respectively. A total of five samples were assigned to a genetic reporting group with less than 90% confidence, and two samples had elevated *z*‐scores. Therefore, these samples were dropped from downstream analyses and resulted in 457 individuals in the analysis that were split into 13 mixture collection groups based on their sampled location (Table 2).

**FIGURE 2:**
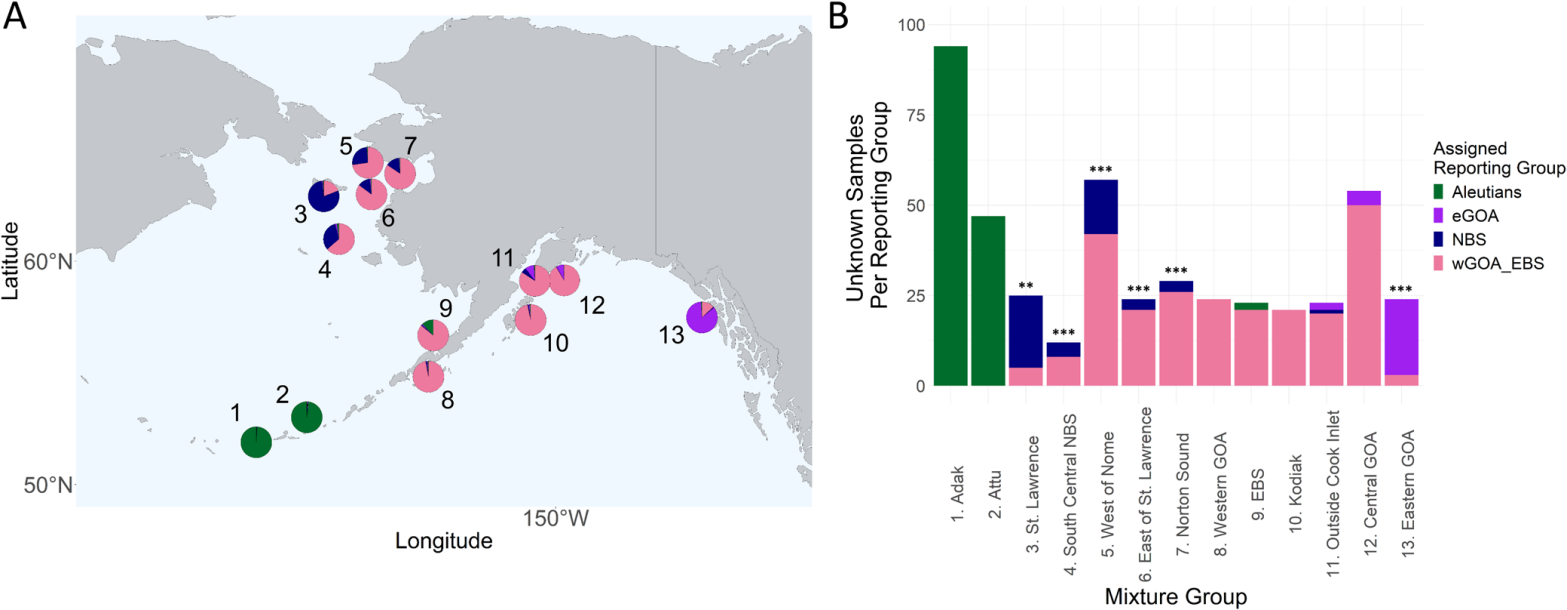
Stock compositions of adult Pacific cod caught in summer. (A) mixed stock proportions of each genetic reporting group for mixture collections 1–13 and (B) corresponding individual assignment based on mixture analysis. Significance values on panel B indicate whether the summer mixture collection differed from the local winter spawning group using a Chi‐square test. Blank *p* > 0.05, **p < 0.01, ***p < 0.001.

Assessment of the seasonal change in reporting group proportions between the winter spawning season and the summer non‐spawning season uncovered variable results. Within the Aleutian Islands, all samples showed 100% assignment back to the Aleutian Islands reporting group (> 96% mixing proportion), resulting in no significant seasonal change in reporting group proportions (Figure 2; Table 5; Table S1). In the NBS (sample stations 3–7), individuals were assigned to either the NBS or wGOA/EBS reporting group, but the ratio of each reporting group varied across mixture collections with an eastward decline in the mixing proportion of NBS assigned individuals (Figure 2; Table S1). When compared to the winter spawning season, there was a significant change in reporting group proportions in all NBS mixture collections (*p* < 0.002 for St. Lawrence and *p* < 0.0002 for all other NBS collections; Table 5). In the EBS, 88.8% of the mixing proportion belonged to the wGOA/EBS and 9.1% assigned to the Aleutian Islands, resulting in no significant seasonal change in reporting group proportions (*p* > 0.1; Table 5; Table S1). In the western and central GOA, almost all individuals were assigned to the wGOA/EBS reporting group, resulting in no significant seasonal change in reporting group proportions across all sampled locations (Table 5). Specifically, the wGOA and Kodiak mixture collections had 100% assignment to the wGOA/EBS reporting group, resulting in mixing proportions > 96% (Figure 2; Table S1). Cook Inlet and the cGOA both had representation from additional reporting groups with a limited number of individuals assigned to the NBS (5.3% in Cook Inlet) and Aleutian Islands (9.6% and 7.7%, respectively; Table S1). Finally, in the eGOA, a limited number of individuals were assigned to the wGOA/EBS, but the majority were assigned to the eGOA. This resulted in a slightly elevated mixing proportion of 13% wGOA/EBS compared to 84.9% eGOA and, therefore, a significant seasonal change in reporting group proportions was detected (*p* < 0.0002; Table 5; Table S1).

**TABLE 5:** Results from Pearson's chi‐square test with Monte Carlo simulated *p*‐value based on 5000 replicates for each summer mixture collection compared to the winter (baseline) collection that corresponds to that mixture collection's region.

Sample site	Winter comparison	Test statistic (*z*)	*p*	Significance threshold^a^
1. Adak	Aleutians	1.61	0.38	NS
2. West Attu	Aleutians	0.81	0.62	NS
3. St. Lawrence	NBS	17.37	0.002	**
4. South Central NBS	NBS	130.49	0.0002	***
5. West of Nome	NBS	551.3	0.0002	***
6. East of St. Lawrence	NBS	397.16	0.0002	***
7. Norton Sound	NBS	462.17	0.0002	***
8. Eastern Bering Sea	wGOA/EBS	0.32	0.76	NS
9. Western GOA	wGOA/EBS	3.17	0.10	NS
10. Kodiak	wGOA/EBS	2.77	0.15	NS
11. Outside Cook Inlet	wGOA/EBS	0.04	1	NS
12. Central GOA	wGOA/EBS	0.55	0.46	NS
13. Eastern GOA	eGOA	282.24	0.0002	***

^a^ NS, not significant *p* > 0.05, **p < 0.01, ***p < 0.001.

### 3.5 Juvenile Sample Assignment

A total of 34 loci were dropped from the analysis of the juvenile samples due to being genotyped in less than 75% of individuals. The resulting total number of individuals was 657 which were split into 12 mixture collection groups based on their sampled location, year of sampling, and sample sizes (Table 3). A total of 14 samples were assigned to a genetic reporting group with less than 90% confidence, and 1 sample had *z*‐scores outside of the expected range, suggesting it came from a population not represented in the reference dataset.

**FIGURE 3:**
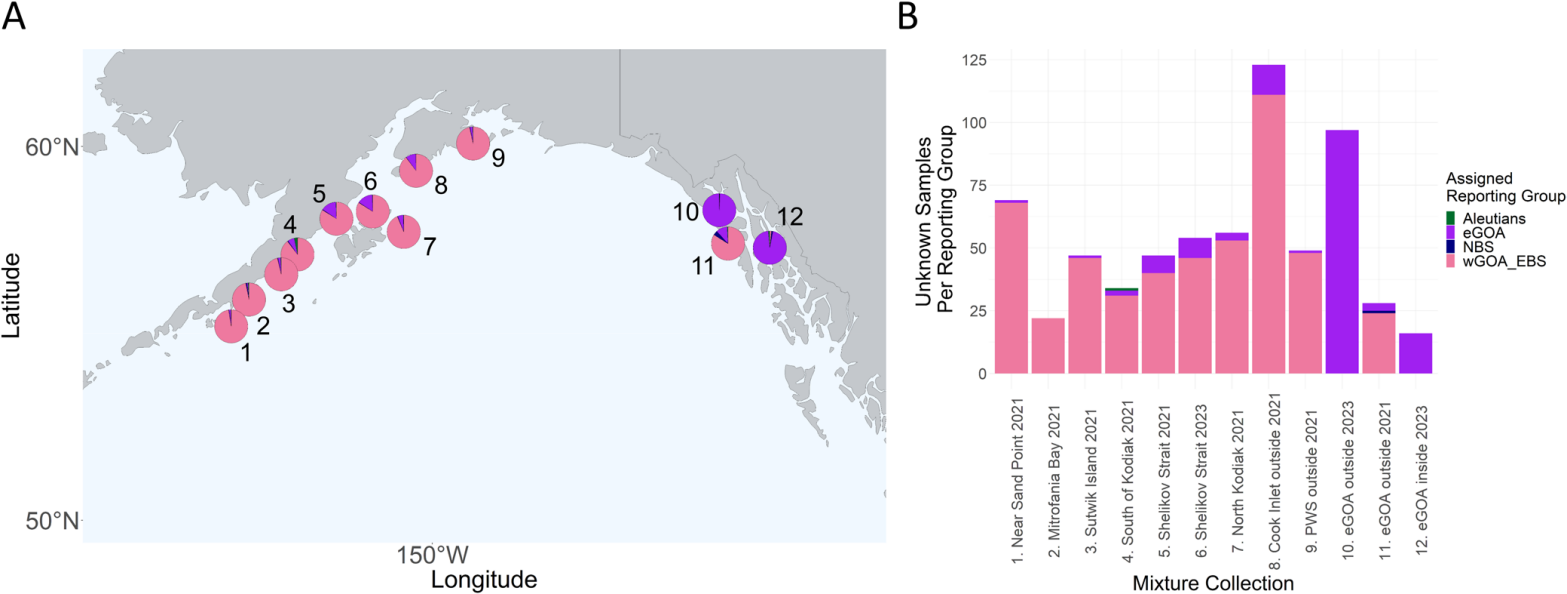
Stock compositions of juvenile Pacific cod samples across the Gulf of Alaska (A) mixed stock proportions of each genetic reporting group for mixture collections 1–12 and (B) corresponding individual assignment based on mixture analysis. Data are filtered for only those individuals assigned with 90% confidence and a *z*‐score of > −5.

Mixture collections in the GOA in both 2021 and 2023 contained almost exclusively eGOA and wGOA/EBS individuals. A general trend of a westward decrease in the proportion of eGOA‐assigned individuals in collections was prevalent when assessing both mixing proportion data (Figure 3A; Table S2) and individual assignments (Figure 3B). There were two notable deviations from this trend, with the first being a majority of wGOA/EBS individuals found in the eGOA in 2021, and second, a slightly increased proportion of eGOA individuals in the Shelikof Strait in both 2021 and 2023 (Figure 3A,B; Table S2).

### 3.6 Juvenile Particle Modeling

**FIGURE 4:**
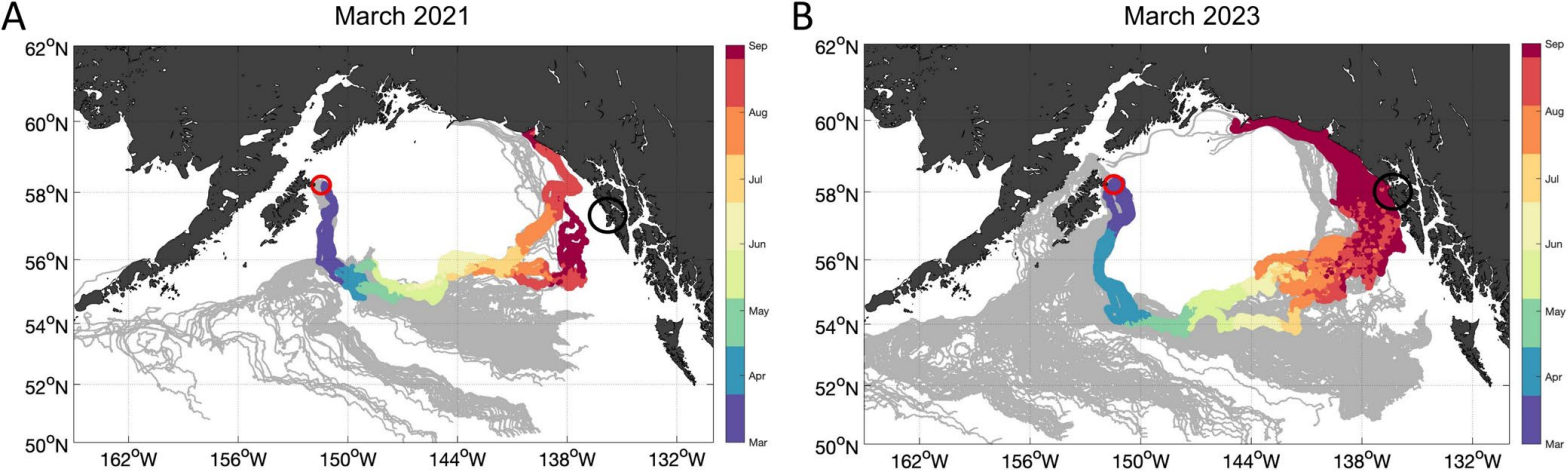
HYCOM model output for a 200‐day simulation of particles released off the northeast corner of Kodiak Island (58.3 N 151.5 W red circle on each map) on (A) March 15, 2021, and (B) March 15, 2023. Black circles off the coast of the eastern Gulf of Alaska (eGOA) represents the juvenile collection points on June 20, 2021, and June 22, 2023. Colored tracks are the particle tracks that were the most likely trajectories to make it to the eGOA with colors representing the month during the simulation and grey tracks are particle releases that had a low probability of reaching the eGOA.

Lagrangian particle integrations for releases on March 15, 2021, and 2023 from the northeast corner of Kodiak Island demonstrated variable particle trajectories with some moving westward; but 94% and 72% of particles were transported off‐shelf into the GOA Gyre in 2021 and 2023, respectively (Figure 4). Of those particles, 3% in 2021 and 23% in 2023 made it to the eGOA by the end of the model run. This eastward trajectory was not found in model runs for 2022, where all particles were transported westward (Figure S8). The time it took for particles to reach the eGOA in 2021 and 2023 differs between years, with the fastest tracks resulting in particles arriving at the eGOA by July–August in 2021 (Figure 4A), but not until September for all tracks in 2023 (Figure 4B). Looking across time for 2021, this eastward movement of particles from Kodiak was prevalent in both February and March, but tapered off in April (Figure S9).

## 4 Discussion

Movement across management areas is a frequent challenge for fisheries management. Applying targeted genetic techniques can be a powerful and inexpensive way for researchers to disentangle the complexities of movement, both seasonally, annually, and across ontogeny. Here, we developed a GT‐seq panel that can identify the genetic stock of origin of Pacific cod with high accuracy. We were able to differentiate four genetically distinct groups present in Pacific cod collected in the Bering Sea and GOA with highly accurate stock assignments: (i) eGOA, (ii) wGOA/EBS, (iii) Aleutian Islands, and (iv) NBS. We then leveraged this panel to investigate movement patterns of two independent datasets: seasonal movement of adult Pacific cod caught throughout the GOA and Bering Sea, and juvenile Pacific cod caught throughout the GOA. Our findings identified little adult seasonal movement in the GOA and Aleutian Islands, but significant movement of two stocks into the NBS, with wGOA/EBS individuals increasing in proportion closer to the Alaska coast. Interestingly, for the juvenile data, we found an overall trend of westward advection of eGOA individuals into the central and western GOA, but with two key deviations from this trend, which suggest a role of mesoscale processes on transport.

### 4.1 Population Genomics of Pacific Cod and Panel Development

Analysis of Pacific cod population structure in our study broadly supports previous population genetic work on Pacific cod that indicated a general signal of isolation by distance (Drinan et al. 2018). However, our high‐resolution lcWGS data revealed a few major genetic breaks in the population structure that both corroborated previous work and allowed us to identify a previously unknown break. Firstly, a genetic break previously hypothesized (Drinan et al. 2018) was confirmed between the eGOA and the wGOA somewhere near Yakutat, suggesting that there may be a significant barrier to gene flow between these two regions, which has been reported for other species (Mueter and Norcross 2002; Palof et al. 2011). Second, the Aleutian Islands were confirmed as a unique population (Spies et al. 2022) that was not fully evident when looking across the whole genome, but was clearly identified when looking at the first two PC axes for highly differentiated markers in the genome. Finally, a new, previously unknown genetic break was found between the wGOA/EBS and the NBS.

Interestingly, the EBS and the wGOA were genetically indistinguishable despite these being separate management units (Barbeaux et al. 2023; Hulson et al. 2023). These two regions have considerable genomic overlap, suggesting that there are high migration rates and/or that divergence is slow due to large population sizes. Recent tagging studies substantiate these findings by observing Unimak Pass (also referred to as “cod alley”) as a major migration pathway between the two basins (J. Nielsen, AFSC, *pers. comm*.) for Pacific cod.

### 4.2 Adult Seasonal Movement

A growing body of evidence suggests that Pacific cod exhibit spawning‐site fidelity, and that some individuals perform long‐distance migrations to productive feeding grounds outside of the spawning season (Rand et al. 2014; Shimada and Kimura 1994). Our results corroborate this evidence by identifying variable seasonal movement of Pacific cod adults across all genetic stocks in the GOA and Bering Sea. Adult seasonal movement patterns ranged from no evidence of movement in some regions to complete mixing of two genetic stocks in others. Tagging studies have also identified variation in movement patterns with some individuals traversing great distances (> 1000 km), while others exhibit little to no movement (Bryan et al. 2021; Rand et al. 2014; Shimada and Kimura 1994), suggesting Pacific cod use a partial migration strategy (Bryan et al. 2021; individual variation in migration patterns within a population). Taken together, this indicates migration propensity varies significantly across regions for Pacific cod.

The NBS sampling region had the most pronounced mixing of genetic groups of all sampled locations, demonstrating that multiple genetically distinct populations use these feeding grounds in the summer months. Higher proportions of wGOA/EBS individuals were found in the more coastal sampling locations, suggesting coastal northward movement of this genetic stock as summer proceeds, whereas our most westward site, St. Lawrence Island, was dominated by NBS individuals, suggesting eastward movement. One hypothesis for this finding is that as the Bering Sea cold pool retreats in the summer, Pacific cod most likely follow prey items into the cold, productive waters of the NBS from multiple directions (Kotwicki et al. 2005; Rand et al. 2014; Stevenson and Lauth 2019). The Bering Sea has experienced continued warming with a higher prevalence of marine heatwaves than in the past (Carvalho et al. 2021) causing the cold pool to completely disappear in some years. This may cause significant shifts or disruptions to Pacific cod movement and the seasonal phenology in this region.

Little to no movement was detected in all other sampled regions within the GOA and Aleutian Islands. Little is known about the movement patterns of adult Pacific cod between the western and eastern GOA, with much of the research focus being on movement in the wGOA and Bering Sea (Bryan et al. 2021; Rand et al. 2014; Shimada and Kimura 1994), but our data suggest movement is limited. Conversely, our Aleutian Islands data support previous tagging studies in groundfish species, including Pacific cod and Pacific halibut ( *Hippoglossus stenolepis* ), which outline extremely limited movement in the Aleutian Islands (Bryan et al. 2021; Loher 2022). The underlying mechanism for this lack of movement remains relatively unknown, but may be influenced by the strong current dynamics that occur in the Aleutian Islands, where the fast‐moving ACC shifts northward (Ladd et al. 2005). Further work evaluating fine‐scale environmental conditions and ecosystem dynamics would be needed to gain a mechanistic understanding of movement in this region.

### 4.3 Juvenile Movement

We observed westward transport of juveniles from the eGOA into the wGOA/EBS, which gradually tapered off as collections moved farther west, lending credence to an overall westward transport in the GOA. Larval projection models (Hinckley et al. 2019) and buoy deployments (Ladd et al. 2005) have offered support for larval movement being predominantly influenced by westward advection within the Alaska Current. Our study also identified two key deviations from this overall trend: (i) a higher‐than‐expected proportion of eGOA juveniles within Shelikof Strait that was consistent across years, and (ii) eastward movement of wGOA/EBS juveniles into the eGOA, which was variable across years. Various mesoscale processes may help shed light on why these deviations are present.

One hypothesis that may explain why we see an increased amount of eGOA individuals in Shelikof Strait is that the strength of the ACC was strong and consistent in our collection years (2021 and 2023), therefore transporting more juveniles than would be expected into Shelikof Strait. The ACC splits off from the Alaska Current into the Shelikof Strait (Rovegno et al. 2009) and this coastal current reaches extremely strong flow rates of up to 100 cm s^−1^ (Bailey et al. 2003), with strong seasonal variation and twice the transport rates in the winter (Stabeno et al. 2016) coincident with Pacific cod spawn timing. Variations in these flow rates impact levels of recruitment in various species, including gadids within the Shelikof Strait (Wilson and Laman 2021). Although our models identified transport of Kodiak individuals into the Shelikof Strait in 2023, but not in 2021, we limited our analysis to a single release site. More data spanning multiple years as well as correlation with oceanographic modeling of multiple release locations would be needed to evaluate the persistence of transport into the Shelikof Strait.

The prevalence of wGOA/EBS individuals in the eGOA in one year, but not the other, suggests that annually varying, smaller‐scale oceanographic processes may create stochastic variation differing from generally observed patterns. Within the GOA, eddies that cause cyclonic movement of water opposite to that of the predominant current are a common occurrence (Henson and Thomas 2008; Xiu et al. 2012). However, their temporal distribution, size, and magnitude in strength vary from year to year. Drifters released in one such eddy (i.e., the Yakutat eddy) resulted in variable final trajectories with a minority of drifters transported back east (Hinckley et al. 2019). Here, we expand on that work and show that an oceanographic general circulation model seeded with passive particles (i.e., “larvae”) from northeast Kodiak resulted in a pronounced signal of advection off‐shelf into the GOA Gyre. Within the GOA gyre, particles were transported to the eGOA, giving a clear trajectory supporting our observed data. Interestingly, we found this oceanographic phenomenon in both 2021 and 2023, even though wGOA/EBS juvenile recruitment was only observed in 2021. One potential explanation for this is that our model produced a path that resulted in faster transport of juveniles to the eGOA in 2021 (i.e., ~July–August) compared to 2023 (i.e., ~September). This indicates that genetic stock‐specific recruitment to the eGOA may be affected by time of sampling, and/or open ocean conditions influencing year‐to‐year survival.

wGOA/EBS recruitment into the eGOA may have significant impacts on population dynamics in Pacific cod. If they survive and reproduce in this region, the influx of wGOA/EBS individuals may impact genetic population structure for the species. Our lcWGS data provide some insight into this by showing relatively strong population differentiation between the wGOA and eGOA, suggesting Pacific cod most likely exhibit natal homing. Therefore, if wGOA/EBS juveniles do survive, they most likely return to their natal sites for spawning. Further work with yearly sampling of this recruitment cohort would be necessary to evaluate how long this signal of wGOA/EBS individuals persists as fish develop.

### 4.4 Management Implications

Our study outlined four genetically distinct stocks within Alaska waters, whereas management of Pacific cod currently recognizes three stocks: the GOA (Hulson et al. 2023), the Bering Sea (Barbeaux et al. 2023), and the Aleutian Islands (Spies et al. 2022). Our findings broadly support these management groups with a few caveats. Firstly, we identified two genetically distinct groups within the GOA, a western and an eastern GOA group, that had been hypothesized previously (Drinan et al. 2018) and were confirmed in our study. Second, current management of Pacific cod considers the Bering Sea to represent a single stock, but our results show that two genetically distinct populations occupy these waters in the summer and are mixing to some extent on the spawning grounds themselves (i.e., two genetic stocks found within the Pervenets Canyon spawning aggregation). Commercial trawl fishing of Pacific cod in the Bering Sea occurs in the winter months when Pacific cod are typically on the spawning grounds. Longline fishing vessels have shown movement into NBS waters (where trawling is not permitted) in years when the cold pool recedes. With the higher prevalence of marine heatwaves in the Bering Sea, these grounds may be utilized more often by fishing vessels, resulting in commercial fishing occurring on two genetically distinct stocks, both on the spawning grounds and in the NBS. Finally, our data identified no genetic differentiation between the wGOA and EBS, which are currently managed as separate stocks. This aligns with previous tagging data indicating that cod are moving between these regions. Taken together, our results can be used to assist managers and stock assessment scientists in decision‐making for these commercially important stocks of Pacific cod.

### 4.5 Overall Conclusions and Broader Implications Beyond Pacific Cod

Here, we provided both the most detailed population genetic assessment of Pacific cod to date and used those data to develop a new, inexpensive genetic tool that can be leveraged to better understand the ecology of Pacific cod. Our initial use of this tool provides novel insights into both genetic stock‐specific recruitment patterns and seasonal migration patterns, which outline the dynamic nature of Pacific cod movement. Our findings enable many new and interesting lines of inquiry for future research including: (i) how consistent is adult seasonal movement from year to year?, (ii) does stock‐specific movement in the NBS vary based on yearly variations in sea‐ice extent?, (iii) do juveniles survive in the GOA Gyre and would this be a plausible trajectory for recruitment of wGOA/EBS juveniles to the eGOA?, and (iv) if wGOA/EBS individuals do survive transport across the GOA to the eGOA, do they reproduce in the eGOA or do they either perish or return to their natal spawning sites as mature adults? In addition to the questions outlined above, the availability of the GT‐seq panel will make large‐scale projects more feasible and, therefore, will facilitate a range of research opportunities previously limited due to the cost of sequencing.

Marine fishes are important targets for genetic tool developments due to their commercial and ecological importance, difficulty to track due to long‐distance mobility at multiple life stages, and spatiotemporal variability in recruitment success. However, many marine fishes, including Pacific cod, exhibit high levels of gene flow, resulting in low genetic structure. This low structure has historically precluded GSI (Araujo et al. 2014), but here, we show that a high‐resolution panel designed from whole‐genome sequencing data can enable accurate GSI even when genetic structure is low (see also: Beemelmanns et al. 2025; Beck et al. 2025). Because of this and their low cost to implement, GT‐seq panels designed using an initial set of whole‐genome sequencing data represent an efficient, affordable, and accurate tool for evaluating GSI in low genetically structured populations.

In particular, targeted GT‐seq panels could provide useful information on the movement of marine fish that is difficult or expensive to obtain with physical tags. Movement is an important parameter in many spatial stock assessments that are used to manage large marine fisheries (Cadrin et al. 2023). Much of the movement data that are integrated into stock assessments is from tags; for example, movement matrices for sablefish ( *Anoplopoma fimbria* ) have been developed from multidecade tagging efforts that have deployed hundreds of thousands of tags (Hanselman et al. 2014). However, tagging requires significant investment in terms of personnel time and capital. In many situations, it is likely that GSI with a GT‐seq panel would provide more robust movement data at a lower cost compared to tagging. Some examples of fisheries where this tool could potentially be applied to improve stock assessments include Pacific Ocean Perch ( *Sebastes alutus* ) in Alaska (Kapur et al. 2023 POP), walleye pollock ( *Gadus chalcogrammus* ) in the Bering Sea and GOA (Levine et al. 2024), and Pacific halibut ( *Hippoglossus stenolepis* ) along the west coast of the United States and Canada (Webster et al. 2013).

Additionally, when available, combining results from tagging data and genetics is an important step forward for understanding movement. A major strength of tagging data is the resulting high‐resolution, individual‐level movement data that can be used to evaluate movement patterns both within and between genetic stocks. GSI broadens our understanding of population‐level dynamics on a large scale, but with the limitation that the resolution is only to the level of genetically distinct stocks and cannot identify within‐stock movement. By incorporating both data types in future studies, we will have a powerful ability to delineate movement patterns in highly mobile fish species.

## Conflicts of Interest

The authors declare no conflicts of interest.

## Supporting information


**Data S1:** eva70174‐sup‐0001‐Supinfo.pdf.

## Data Availability

Upon acceptance of the manuscript, the data that support the findings of this study will be available in the Sequence Read Archive (SRA), which is accessible from the National Center for Biotechnology Information (NCBI). All code and data files used in the analysis of this study will be published and available on GitHub.

## References

[eva70174-bib-0001] Abelman, A. , M. Dalton , R. Dame , et al. 2023. “Stock Assessment and Fishery Evaluation Report for the Groundfish Fisheries for the Gulf of Alaska and Bering Sea/Aleutian Islands Area: Economic Status of the Groundfish Fisheries Off Alaska.” North Pacific Fishery Management Council 1007 West 3rd Ave., Suite 400, L92 Building, 4th Floor, Anchorage, AK 99501.

[eva70174-bib-0002] Anderson, E. C. , A. J. Clemento , M. A. Campbell , et al. 2025. “A Multipurpose Microhaplotype Panel for Genetic Analysis of California Chinook Salmon.” Evolutionary Applications 18, no. 5: e70110. 40365168 10.1111/eva.70110 PMC12070256

[eva70174-bib-0003] Anderson, E. C. , R. S. Waples , and S. T. Kalinowski . 2008. “An Improved Method for Predicting the Accuracy of Genetic Stock Identification.” Canadian Journal of Fisheries and Aquatic Sciences 65, no. 7: 1475–1486. 10.1139/F08-049.

[eva70174-bib-0004] Araujo, H. A. , J. R. Candy , T. D. Beacham , B. White , and C. Wallace . 2014. “Advantages and Challenges of Genetic Stock Identification in Fish Stocks With Low Genetic Resolution.” Transactions of the American Fisheries Society 143, no. 2: 479–488. 10.1080/00028487.2013.855258.

[eva70174-bib-0005] Baetscher, D. S. , A. J. Clemento , T. C. Ng , E. C. Anderson , and J. C. Garza . 2018. “Microhaplotypes Provide Increased Power From Short‐Read DNA Sequences for Relationship Inference.” Molecular Ecology Resources 18, no. 2: 296–305. 10.1111/1755-0998.12737. 29143457

[eva70174-bib-0006] Bailey, K. M. , E. S. Brown , and J. T. Duffy‐Anderson . 2003. “Aspects of Distribution, Transport and Recruitment of Alaska Plaice ( *Pleuronectes quadrituberculatus* ) in the Gulf of Alaska and Eastern Bering Sea: Comparison of Marginal and Central Populations.” Journal of Sea Research 50, no. 2–3: 87–95. 10.1016/S1385-1101(03)00064-9.

[eva70174-bib-0007] Barbeaux, S. J. , L. Barnett , M. Hall , et al. 2023. “Assessment of the Pacific Cod Stock in the Eastern Bering Sea.” North Pacific Fishery Management Council, 1007 West 3rd Ave., Suite 400, L92 Building, 4th Floor, Anchorage, AK 99501.

[eva70174-bib-0008] Beck, J. N. , D. S. Baetscher , C. Tobin , et al. 2025. “Quantifying Impacts of Seabird Bycatch Using Genetic Assignment: A Case Study of Black‐Footed Albatross in U.S. Fisheries.” Biological Conservation 303: 110965. 10.1016/j.biocon.2025.110965.

[eva70174-bib-0009] Beemelmanns, A. , R. Bouchard , S. Michaelides , et al. 2025. “Development of SNP Panels From Low‐Coverage Whole Genome Sequencing (lcWGS) to Support Indigenous Fisheries for Three Salmonid Species in Northern Canada.” Molecular Ecology Resources 25, no. 3: e14040. 39552382 10.1111/1755-0998.14040 PMC11887602

[eva70174-bib-0010] Bonanomi, S. , N. O. Therkildsen , A. Retzel , et al. 2016. “Historical DNA Documents Long Distance Natal Homing in Marine Fish.” Molecular Ecology 25: 2727–2734. 10.1111/mec.13580. 26859133

[eva70174-bib-0011] Bootsma, M. L. , K. M. Gruenthal , G. J. McKinney , et al. 2020. “A GT‐Seq Panel for Walleye ( *Sander vitreus* ) Provides Important Insights for Efficient Development and Implementation of Amplicon Panels in Non‐Model Organisms.” Molecular Ecology Resources 20, no. 6: 1706–1722. 10.1111/1755-0998.13226. 32668508

[eva70174-bib-0012] Broms, C. , W. Melle , and J. K. Horne . 2012. “Navigation Mechanisms of Herring During Feeding Migration: The Role of Ecological Gradients on an Oceanic Scale.” Marine Biology Research 8, no. 5–6: 461–474. 10.1080/17451000.2011.640689.

[eva70174-bib-0013] Bryan, D. R. , S. F. McDermott , J. K. Nielsen , D. Fraser , and K. M. Rand . 2021. “Seasonal Migratory Patterns of Pacific Cod (*Gadus macrocephalus*) in the Aleutian Islands.” Animal Biotelemetry 9, no. 24: 1–18. 10.1186/s40317-021-00250-2.

[eva70174-bib-0014] Cadrin, S. X. , D. R. Goethel , A. Berger , and E. Jardim . 2023. “Best Practices for Defining Spatial Boundaries and Spatial Structure in Stock Assessment.” Fisheries Research 262: 106650. 10.1016/j.fishres.2023.106650.

[eva70174-bib-0015] Campbell, N. R. , S. A. Harmon , and S. R. Narum . 2015. “Genotyping‐In‐Thousands by Sequencing (GT‐Seq): A Cost Effective SNP Genotyping Method Based on Custom Amplicon Sequencing.” Molecular Ecology Resources 15, no. 4: 855–867. 10.1111/1755-0998.12357. 25476721

[eva70174-bib-0016] Carpi, P. , T. Loher , L. L. Sadorus , et al. 2021. “Ontogenetic and Spawning Migration of Pacific Halibut: A Review.” Reviews in Fish Biology and Fisheries 31, no. 4: 879–908. 10.1007/s11160-021-09672-w.

[eva70174-bib-0017] Carr, S. D. , X. J. Capet , J. C. McWilliams , J. T. Pennington , and F. P. Chavez . 2008. “The Influence of Diel Vertical Migration on Zooplankton Transport and Recruitment in an Upwelling Region: Estimates From a Coupled Behavioral‐Physical Model.” Fisheries Oceanography 17, no. 1: 1–15. 10.1111/j.1365-2419.2007.00447.x.

[eva70174-bib-0018] Carvalho, K. S. , T. E. Smith , and S. Wang . 2021. “Bering Sea Marine Heatwaves: Patterns, Trends and Connections With the Arctic.” Journal of Hydrology 600: 126462. 10.1016/j.jhydrol.2021.126462.

[eva70174-bib-0019] Cunningham, K. M. , M. F. Canino , I. B. Spies , and L. Hauser . 2009. “Genetic Isolation by Distance and Localized Fjord Population Structure in Pacific Cod ( *Gadus macrocephalus* ): Limited Effective Dispersal in the Northeastern Pacific Ocean.” Canadian Journal of Fisheries and Aquatic Sciences 66: 153–166. 10.1139/F08-199.

[eva70174-bib-0020] Dagestad, K.‐F. , J. Röhrs , Ø. Breivik , and B. Ådlandsvik . 2018. “OpenDrift v1.0: A Generic Framework for Trajectory Modeling.” Geoscience Model Development 11, no. 4: 1405–1420. 10.5194/gmd-2017-205.

[eva70174-bib-0021] Drinan, D. P. , K. M. Gruenthal , M. F. Canino , D. Lowry , M. C. Fisher , and L. Hauser . 2018. “Population Assignment and Local Adaptation Along an Isolation‐By‐Distance Gradient in Pacific Cod ( *Gadus macrocephalus* ).” Evolutionary Applications 11, no. 8: 1448–1464. 10.1111/eva.12639. 30151052 PMC6100185

[eva70174-bib-0022] Duffy‐Anderson, J. T. , S. J. Barbeaux , E. Farley , et al. 2016. “The Critical First Year of Life of Walleye Pollock ( *Gadus chalcogrammus* ) in the Eastern Bering Sea: Implications for Recruitment and Future Research.” Deep‐Sea Research Part II: Topical Studies in Oceanography 134: 283–301. 10.1016/j.dsr2.2015.02.001.

[eva70174-bib-0023] Euclide, P. T. , W. A. Larson , Y. Shi , et al. 2023. “Conserved Islands of Divergence Associated With Adaptive Variation in Sockeye Salmon Are Maintained by Multiple Mechanisms.” Molecular Ecology 1: 1–21. 10.1111/mec.17126. PMC11628665 37695544

[eva70174-bib-0024] Gillanders, B. M. , K. W. Able , J. A. Brown , D. B. Eggleston , and P. F. Sheridan . 2003. “Evidence of Connectivity Between Juvenile and Adult Habitats for Mobile Marine Fauna: An Important Component of Nurseries.” Marine Ecology Progress Series 247: 281–295. 10.3354/meps247281.

[eva70174-bib-0025] Godø, O. R. , V. Hjellvik , S. A. Iversen , A. Slotte , E. Tenningen , and T. Torkelsen . 2004. “Behaviour of Mackerel Schools During Summer Feeding Migration in the Norwegian Sea, as Observed From Fishing Vessel Sonars.” ICES Journal of Marine Science 61, no. 7: 1093–1099. 10.1016/j.icesjms.2004.06.009.

[eva70174-bib-0026] Goethel, D. R. , K. M. Bosley , D. H. Hanselman , et al. 2019. “Exploring the Utility of Different Tag‐Recovery Experimental Designs for Use in Spatially Explicit, Tag‐Integrated Stock Assessment Models.” Fisheries Research 219: 105320. 10.1016/j.fishres.2019.105320.

[eva70174-bib-0027] Goethel, D. R. , T. J. Quinn , and S. X. Cadrin . 2011. “Incorporating Spatial Structure in Stock Assessment: Movement Modeling in Marine Fish Population Dynamics.” Reviews in Fisheries Science 19, no. 2: 119–136. 10.1080/10641262.2011.557451.

[eva70174-bib-0028] Hanselman, D. H. , J. Heifetz , K. B. Echave , and S. C. Dressel . 2014. “Move It or Lose It: Movement and Mortality of Sablefish Tagged in Alaska.” Canadian Journal of Fisheries and Aquatic Sciences 72, no. 2: 238–251. 10.1139/cjfas-2014-0251.

[eva70174-bib-0029] Henson, S. A. , and A. C. Thomas . 2008. “A Census of Oceanic Anticyclonic Eddies in the Gulf of Alaska.” Deep‐Sea Research Part I: Oceanographic Research Papers 55, no. 2: 163–176. 10.1016/j.dsr.2007.11.005.

[eva70174-bib-0030] Hinckley, S. , W. T. Stockhausen , K. O. Coyle , et al. 2019. “Connectivity Between Spawning and Nursery Areas for Pacific Cod ( *Gadus macrocephalus* ) in the Gulf of Alaska.” Deep‐Sea Research Part II: Topical Studies in Oceanography 165: 113–126. 10.1016/j.dsr2.2019.05.007.

[eva70174-bib-0031] Huebert, K. B. , R. K. Cowen , and S. Sponaugle . 2011. “Vertical Migrations of Reef Fish Larvae in the Straits of Florida and Effects on Larval Transport.” Limnology and Oceanography 56, no. 5: 1653–1666. 10.4319/lo.2011.56.5.1653.

[eva70174-bib-0032] Hulson, P.‐J. F. , S. J. Barbeaux , B. Ferriss , et al. 2023. “Assessment of the Pacific Cod Stock in the Gulf of Alaska.” North Pacific Fishery Management Council, 1007 West 3rd Ave., Suite 400, L92 Building, 4th Floor, Anchorage, AK 99501.

[eva70174-bib-0033] Hurst, T. P. , B. J. Laurel , and L. Ciannelli . 2010. “Ontogenetic Patterns and Temperature‐Dependent Growth Rates in Early Life Stages of Pacific Cod (*Gadus macrocephalus*).” Fishery Bulletin 108, no. 4: 382–392.

[eva70174-bib-0034] Kapur, M. S. , P.‐J. Hulson , and B. C. Williams . 2023. “Assessment of the Pacific Ocean Perch Stock in the Gulf of Alaska.” North Pacific Fishery Management Council, Anchorage, AK.

[eva70174-bib-0035] Kidd, K. K. , A. J. Pakstis , W. C. Speed , et al. 2013. “Microhaplotype Loci Are a Powerful New Type of Forensic Marker.” Forensic Science International: Genetics Supplement Series 4, no. 1: e123–e124.

[eva70174-bib-0036] Kotwicki, S. , T. W. Buckley , T. Honkalehto , and G. Walters . 2005. “Variation in the Distribution of Walleye Pollock (*Theragra chalcogramma*) With Temperature and Implications for Seasonal Migration.” Fishery Bulletin 103, no. 4: 574–587.

[eva70174-bib-0037] Ladd, C. , G. L. Hunt , C. W. Mordy , S. A. Salo , and P. J. Stabeno . 2005. “Marine Environment of the Eastern and Central Aleutian Islands.” Fisheries Oceanography 14: 22–38. 10.1111/j.1365-2419.2005.00373.x.

[eva70174-bib-0038] Larson, W. A. , L. W. Seeb , M. V. Everett , R. K. Waples , W. D. Templin , and J. E. Seeb . 2014. “Genotyping by Sequencing Resolves Shallow Population Structure to Inform Conservation of Chinook Salmon ( *Oncorhynchus tshawytscha* ).” Evolutionary Applications 7, no. 3: 355–369. 10.1111/eva.12128. 24665338 PMC3962296

[eva70174-bib-0039] Laurel, B. J. , T. P. Hurst , L. A. Copeman , and M. W. Davis . 2008. “The Role of Temperature on the Growth and Survival of Early and Late Hatching Pacific Cod Larvae ( *Gadus macrocephalus* ).” Journal of Plankton Research 30, no. 9: 1051–1060. 10.1093/plankt/fbn057.

[eva70174-bib-0040] Levine, R. M. , A. De Robertis , C. Bassett , M. Levine , and J. N. Ianelli . 2024. “Acoustic Observations of Walleye Pollock ( *Gadus chalcogrammus* ) Migration Across the US‐Russia Boundary in the Northwest Bering Sea.” ICES Journal of Marine Science 81, no. 6: 1111–1125. 10.1093/icesjms/fsae071.

[eva70174-bib-0041] Loher, T. 2022. “Dispersal and Seasonal Movements of Pacific Halibut (*Hippoglossus stenolepis*) in the Eastern Bering Sea and Aleutian Islands, as Inferred From Satellite‐Transmitting Archival Tags.” Animal Biotelemetry 10, no. 18: 1–21. 10.1186/s40317-022-00288-w.

[eva70174-bib-0042] Lowerre‐Barbieri, S. K. , C. Friess , L. P. Griffin , et al. 2021. “Movescapes and Eco‐Evolutionary Movement Strategies in Marine Fish: Assessing a Connectivity Hotspot.” Fish and Fisheries 22, no. 6: 1321–1344. 10.1111/faf.12589.

[eva70174-bib-0043] Moran, B. M. , and E. C. Anderson . 2019. “Bayesian Inference From the Conditional Genetic Stock Identification Model.” Canadian Journal of Fisheries and Aquatic Sciences 76, no. 4: 551–560. 10.1139/cjfas-2018-0016.

[eva70174-bib-0045] Mueter, F. J. , and B. L. Norcross . 2002. “Spatial and Temporal Patterns in the Demersal Fish Community on the Shelf and Upper Slope Regions of the Gulf of Alaska.” Fishery Bulletin 100, no. 3: 559–581.

[eva70174-bib-0046] Palof, K. J. , J. Heifetz , and A. J. Gharrett . 2011. “Geographic Structure in Alaskan Pacific Ocean Perch ( *Sebastes alutus* ) Indicates Limited Lifetime Dispersal.” Marine Biology 158, no. 4: 779–792. 10.1007/s00227-010-1606-2.

[eva70174-bib-0048] Perry, A. L. , P. J. Low , J. R. Ellis , and J. D. Reynolds . 2005. “Climate Change and Distribution Shifts in Marine Fishes.” Science 308, no. 5730: 1912–1915. 10.1126/science.1111322. 15890845

[eva70174-bib-0049] R Core Team . 2016. “R: A Language and Environment for Statistical Computing.” R Foundation for Statistical Computing. Vienna, Austria.

[eva70174-bib-0050] Rand, K. M. , P. Munro , S. K. Neidetcher , and D. G. Nichol . 2014. “Observations of Seasonal Movement From a Single Tag Release Group of Pacific Cod in the Eastern Bering Sea.” Marine and Coastal Fisheries 6, no. 1: 287–296. 10.1080/19425120.2014.976680.

[eva70174-bib-0051] Rovegno, P. S. , C. A. Edwards , and K. W. Bruland . 2009. “Observations of a Kenai Eddy and a Sitka Eddy in the Northern Gulf of Alaska.” Journal of Geophysical Research: Oceans 114, no. C11012: 1–18. 10.1029/2009JC005451.

[eva70174-bib-0052] Shimada, A. M. , and D. K. Kimura . 1994. “Seasonal Movements of Pacific Cod, *Gadus macrocephalus*, in the Eastern Bering Sea and Adjacent Waters Based on Tag‐Recapture Data.” Fishery Bulletin 92, no. 4: 800–816.

[eva70174-bib-0053] Spies, I. 2012. “Landscape Genetics Reveals Population Subdivision in Bering Sea and Aleutian Islands Pacific Cod.” Transactions of the American Fisheries Society 141, no. 6: 1557–1573. 10.1080/00028487.2012.711265.

[eva70174-bib-0054] Spies, I. , D. P. Drinan , E. L. Petrou , et al. 2021. “Evidence for Selection and Spatially Distinct Patterns Found in a Putative Zona Pellucida Gene in Pacific Cod, and Implications for Management.” Ecology and Evolution 11, no. 23: 16661–16679. 10.1002/ece3.8284. 34938464 PMC8668774

[eva70174-bib-0055] Spies, I. , K. M. Gruenthal , D. P. Drinan , et al. 2020. “Genetic Evidence of a Northward Range Expansion in the Eastern Bering Sea Stock of Pacific Cod.” Evolutionary Applications 13, no. 2: 362–375. 10.1111/eva.12874. 31993082 PMC6976961

[eva70174-bib-0056] Spies, I. , and A. E. Punt . 2015. “The Utility of Genetics in Marine Fisheries Management: A Simulation Study Based on Pacific Cod Off Alaska.” Canadian Journal of Fisheries and Aquatic Sciences 72, no. 9: 1415–1432. 10.1139/cjfas-2014-0050.

[eva70174-bib-0057] Spies, I. , P. D. Spencer , and A. E. Punt . 2015. “Where Do We Draw the Line? A Simulation Approach for Evaluating Management of Marine Fish Stocks With Isolation‐By‐Distance Stock Structure.” Canadian Journal of Fisheries and Aquatic Sciences 72, no. 7: 968–982. 10.1139/cjfas-2014-0366.

[eva70174-bib-0058] Spies, I. , C. Tarpey , T. Kristiansen , M. Fisher , S. Rohan , and L. Hauser . 2022. “Genomic Differentiation in Pacific Cod Using Pool‐Seq.” Evolutionary Applications 15, no. 11: 1907–1924. 10.1111/eva.13488. 36426128 PMC9679252

[eva70174-bib-0059] Stabeno, P. J. , S. Bell , W. Cheng , S. Danielson , N. B. Kachel , and C. W. Mordy . 2016. “Long‐Term Observations of Alaska Coastal Current in the Northern Gulf of Alaska.” Deep‐Sea Research Part II: Topical Studies in Oceanography 132: 24–40. 10.1016/j.dsr2.2015.12.016.

[eva70174-bib-0060] Stevenson, D. E. , and R. R. Lauth . 2019. “Bottom Trawl Surveys in the Northern Bering Sea Indicate Recent Shifts in the Distribution of Marine Species.” Polar Biology 42, no. 2: 407–421. 10.1007/s00300-018-2431-1.

[eva70174-bib-0061] Tamario, C. , J. Sunde , E. Petersson , P. Tibblin , and A. Forsman . 2019. “Ecological and Evolutionary Consequences of Environmental Change and Management Actions for Migrating Fish.” Frontiers in Ecology and Evolution 7: 1–24. 10.3389/fevo.2019.00271.

[eva70174-bib-0062] Therkildsen, N. O. , and S. R. Palumbi . 2017. “Practical Low‐Coverage Genomewide Sequencing of Hundreds of Individually Barcoded Samples for Population and Evolutionary Genomics in Nonmodel Species.” Molecular Ecology Resources 17: 194–208. 10.1111/1755-0998.12593. 27496322

[eva70174-bib-0063] Timm, L. E. , W. A. Larson , A. J. Jasonowicz , and K. M. Nichols . 2024. “Whole Genome Resequencing of Sablefish at the Northern End of Their Range Reveals Genetic Panmixia and Large Putative Inversions.” ICES Journal of Marine Science 81: 1096–1110. 10.1093/icesjms/fsae070/.

[eva70174-bib-0064] Warner, R. R. , and R. K. Cowen . 2002. “Local Retention of Production in Marine Populations: Evidence, Mechanisms, and Consequences.” Bulletin of Marine Science 70: 245–249.

[eva70174-bib-0065] Webster, R. A. , W. G. Clark , B. M. Leaman , and J. E. Forsberg . 2013. “Pacific Halibut on the Move: A Renewed Understanding of Adult Migration From a Coastwide Tagging Study.” Canadian Journal of Fisheries and Aquatic Sciences 70, no. 4: 642–653. 10.1139/cjfas-2012-0371.

[eva70174-bib-0066] Wilson, M. T. , and N. Laman . 2021. “Interannual Variation in the Coastal Distribution of a Juvenile Gadid in the Northeast Pacific Ocean: The Relevance of Wind and Effect on Recruitment.” Fisheries Oceanography 30, no. 1: 3–22. 10.1111/fog.12499.

[eva70174-bib-0067] Xiu, P. , F. Chai , H. Xue , L. Shi , and Y. Chao . 2012. “Modeling the Mesoscale Eddy Field in the Gulf of Alaska.” Deep‐Sea Research Part I: Oceanographic Research Papers 63: 102–117. 10.1016/j.dsr.2012.01.006.

