# Supplementary material for: Genetic Data Reveal Nonlocal Juvenile Recruitment and Variable Seasonal Movement of a Highly Mobile Marine Fish Across Alaska
Source: Evol Appl. 2026 Jan 6;19(1):e70174. doi: 10.1111/eva.70174 (PMC12772979; doi:10.1111/eva.70174)
Supplement: Supplementary file 1 — Data S1: eva70174‐sup‐0001‐Supinfo.pdf. [file EVA-19-e70174-s001.pdf]

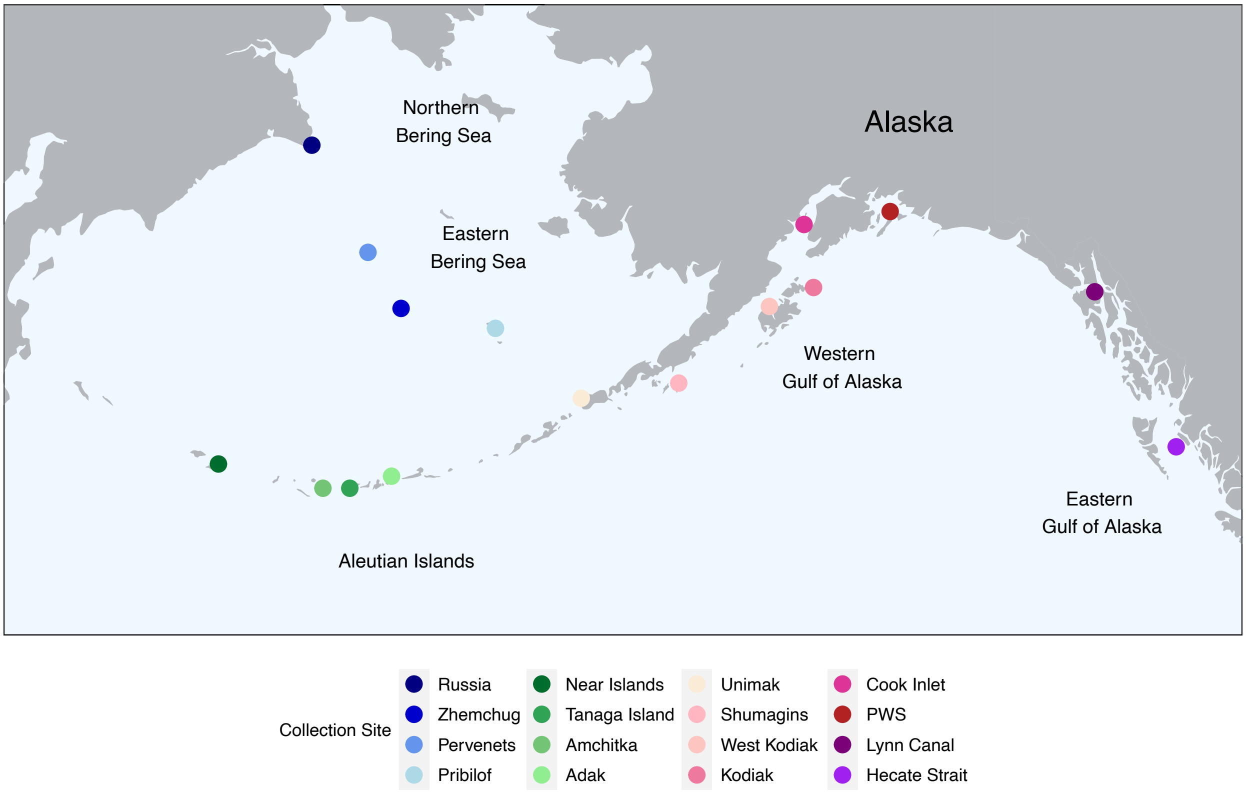

Supplementary Figure 1. Map of sampling sites used in the reference dataset for testing panel performance.

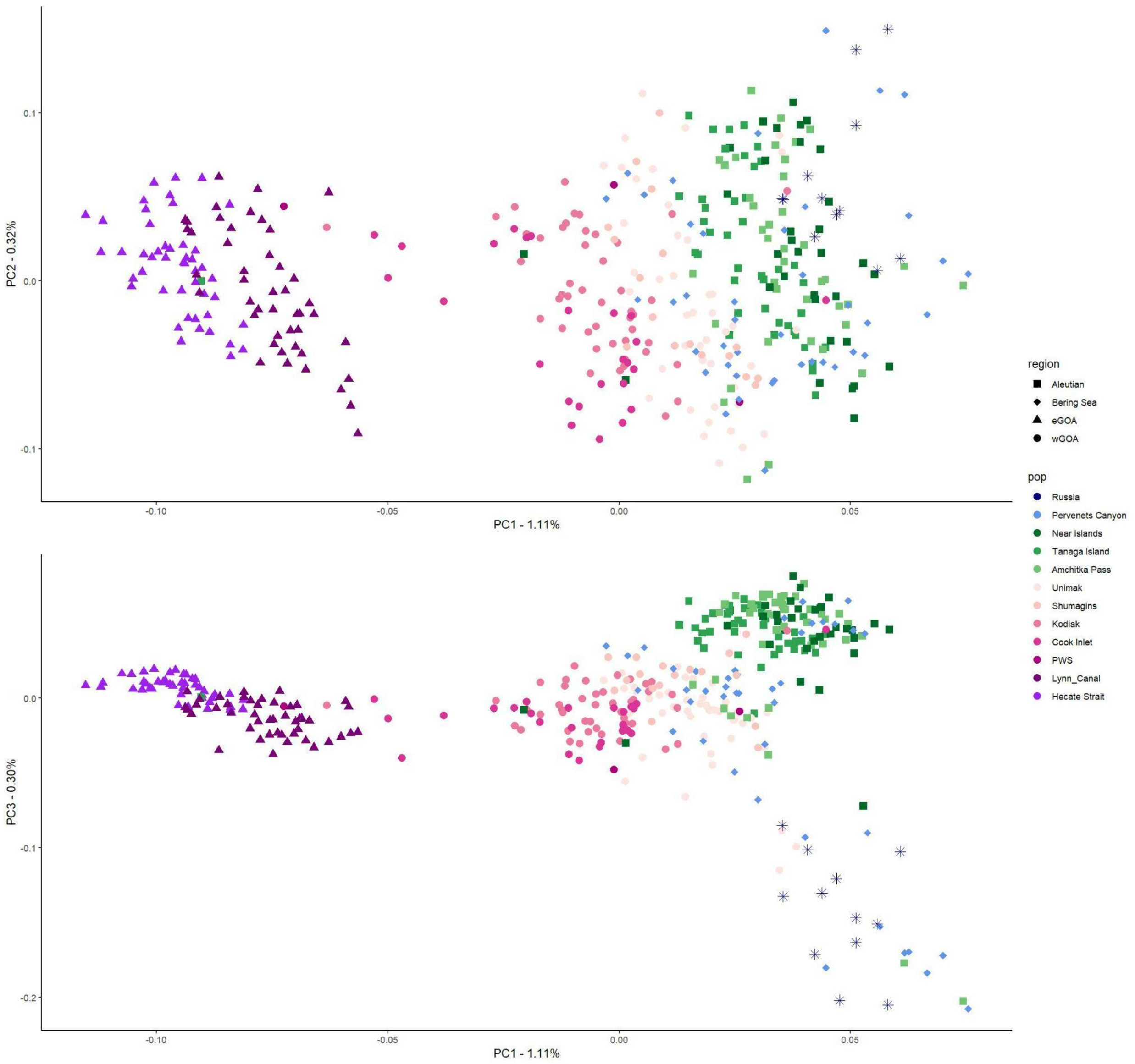

Supplementary Figure 2. Principal component analysis of all SNPs identified with low coverage whole genome sequencing data for PC1 vs. PC2 (top panel) and PC2 vs. PC3 (bottom panel). PC2 represented within sample site variation with PC3 differentiating the NBS. Colors and shapes represent sample sites and regions, respectively.

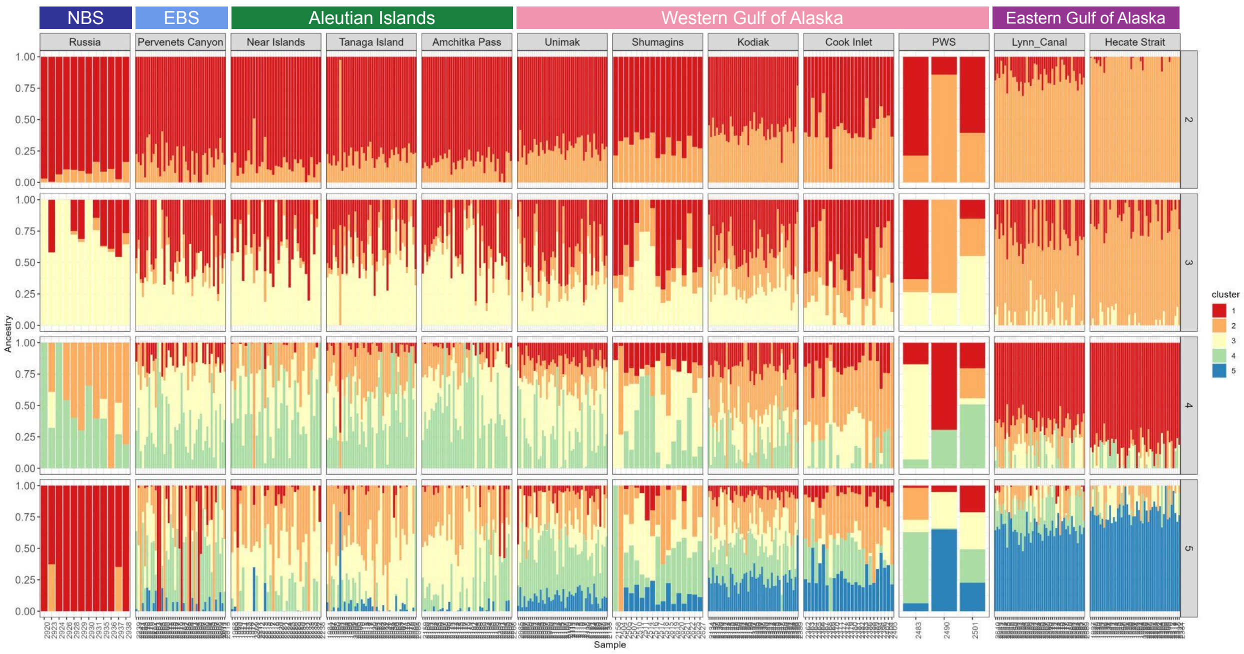

Supplementary Figure 3. Admixture plot for K=2 to 5 where rows represent different values of K and columns represent each individual. Individuals are grouped by their sampling location and the region that location belongs to either the Northern Bering Sea (NBS), Eastern Bering Sea (EBS), Aleutian Islands, Western Gulf of Alaska, or Eastern Gulf of Alaska. Colors within each individual bar represent the proportion of that individuals genome that belongs to a given unique genetic clusters within each admixture run.

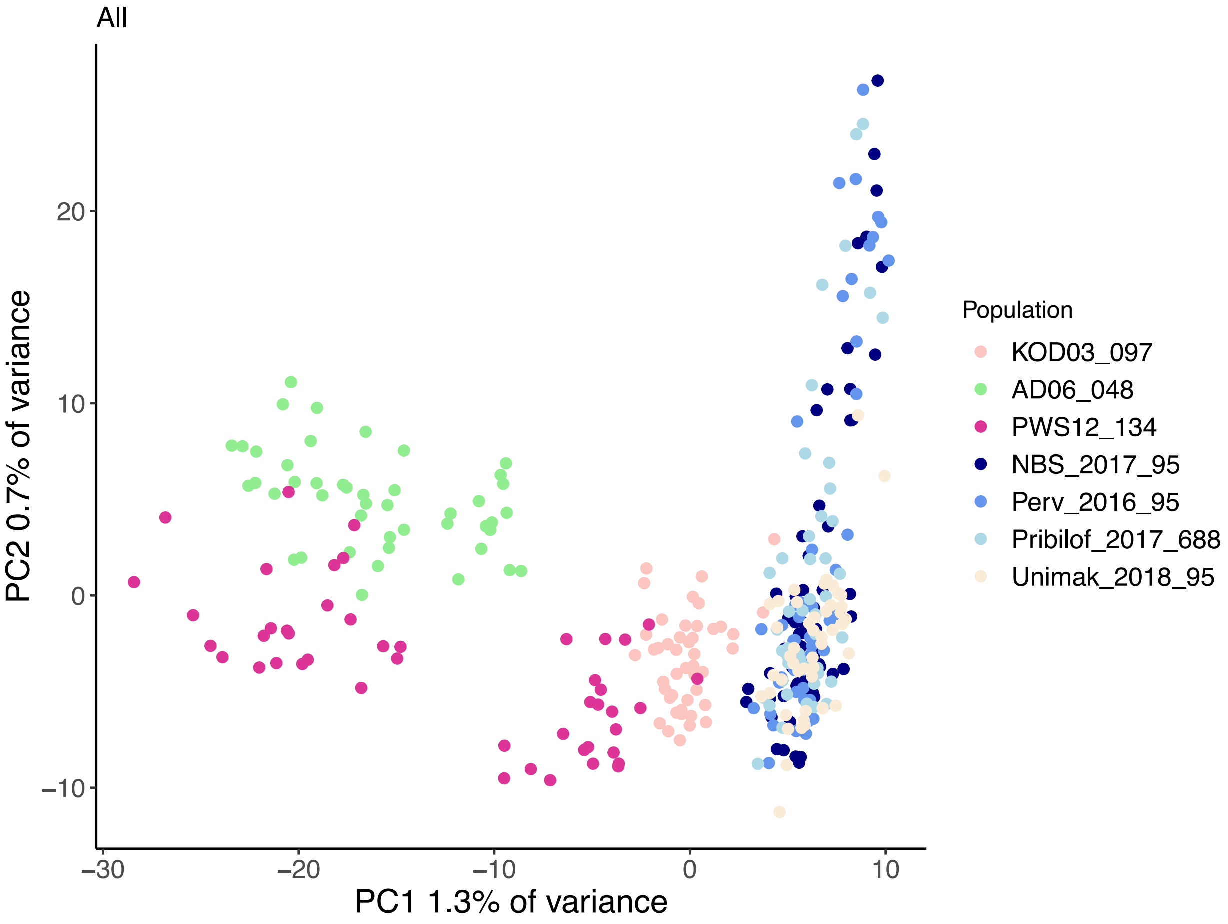

Supplementary Figure 4. Principal Component Analysis of RAD-seq data from Spies et al. 2020 with PC1 plotted against PC2. Colors represent sampled locations.

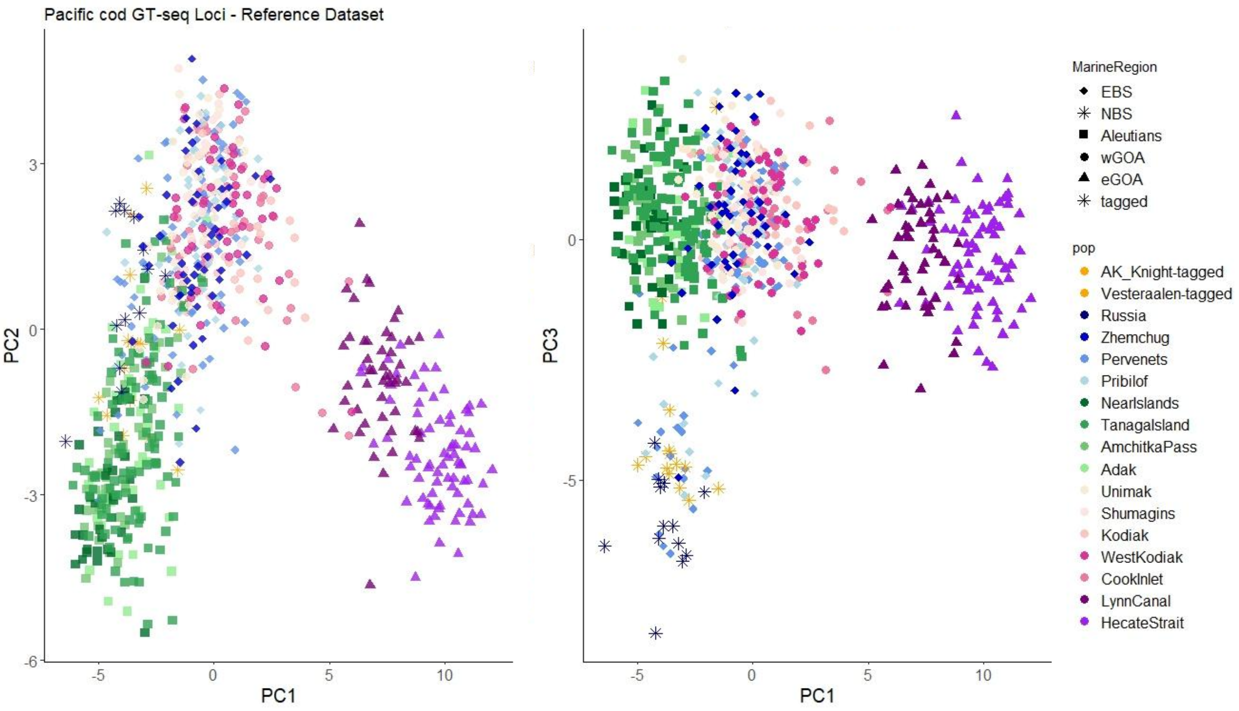

Supplementary Figure 5. Principal component analysis of all individuals from the reference dataset genotyped at all GT-seq loci that passed filtering. Left panel displays PC1 vs. PC2 and the right panel PC1 vs. PC3.

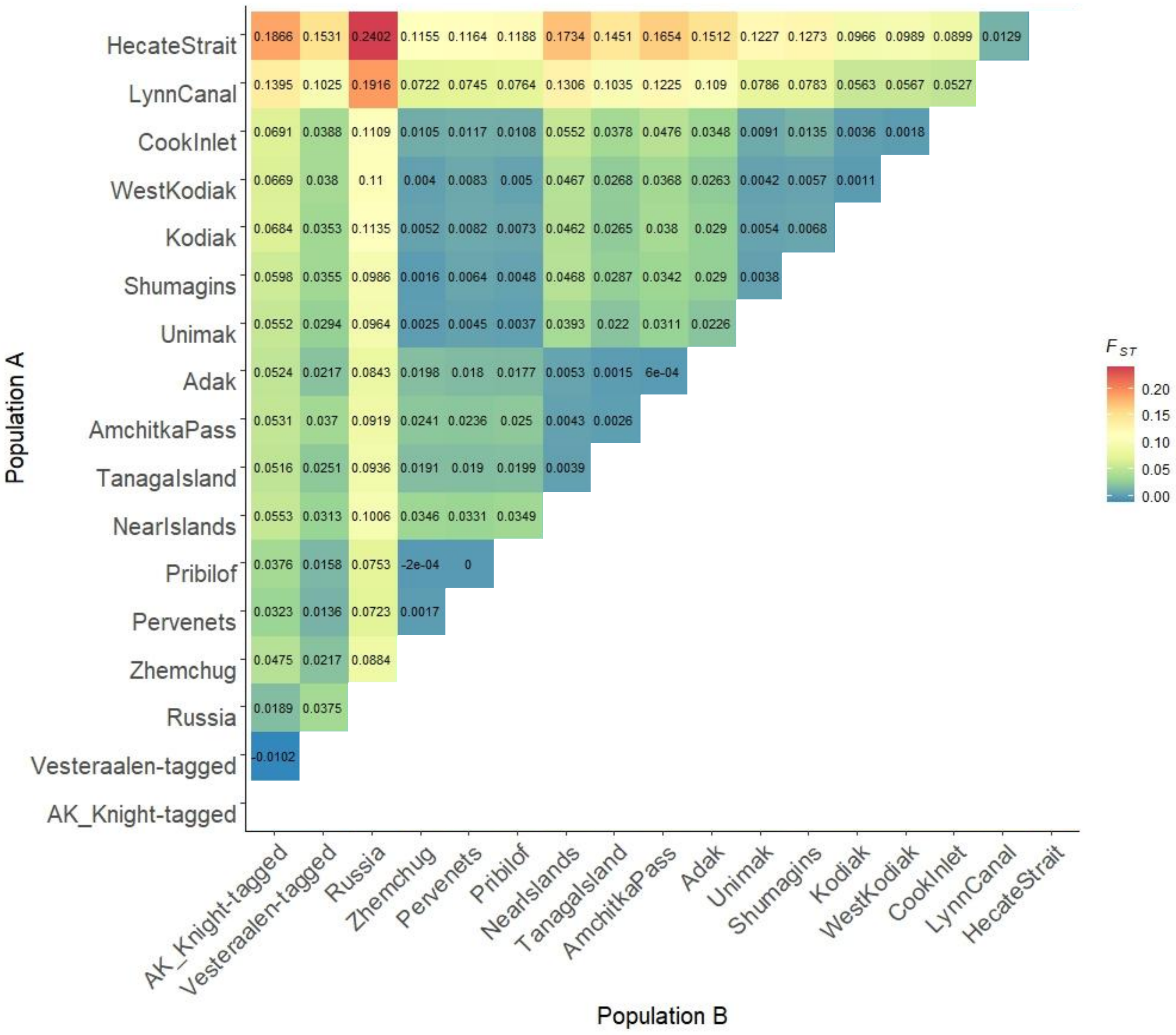

Supplementary Figure 6. Pairwise F_{ST} using GT-seq data across all sampled sites in the reference dataset.

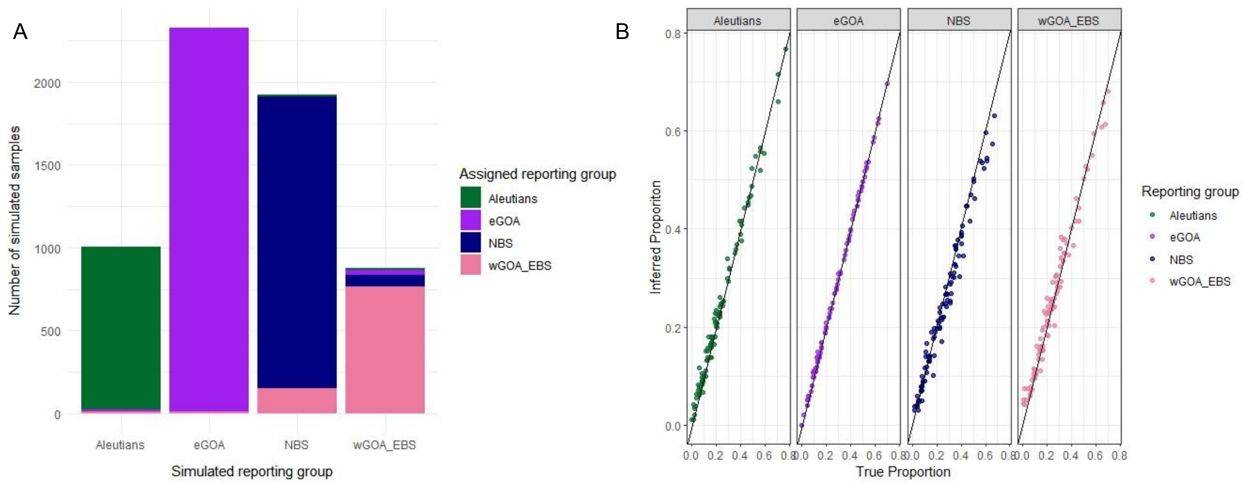

Supplementary Figure 7. GT-seq panel performance based on rubias leave-one-out simulations for samples that were assigned to their respective reporting group with >90% confidence: A) individual assignment to genetic reporting groups, and B) true vs. inferred proportions.

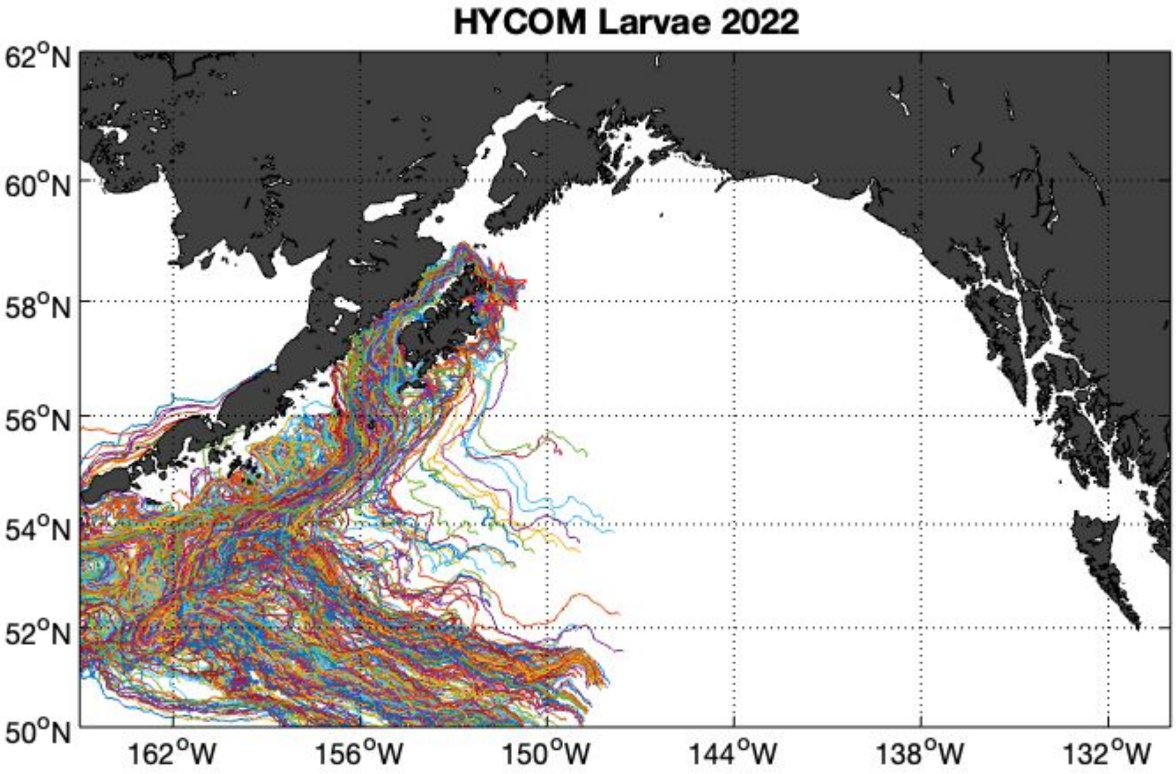

Supplementary Figure 8. HYCOM model output for a 200-day simulation of particles released off the northeast corner of Kodiak Island (58.3 N 151.5 W; red star on map) on March 15, 2022.

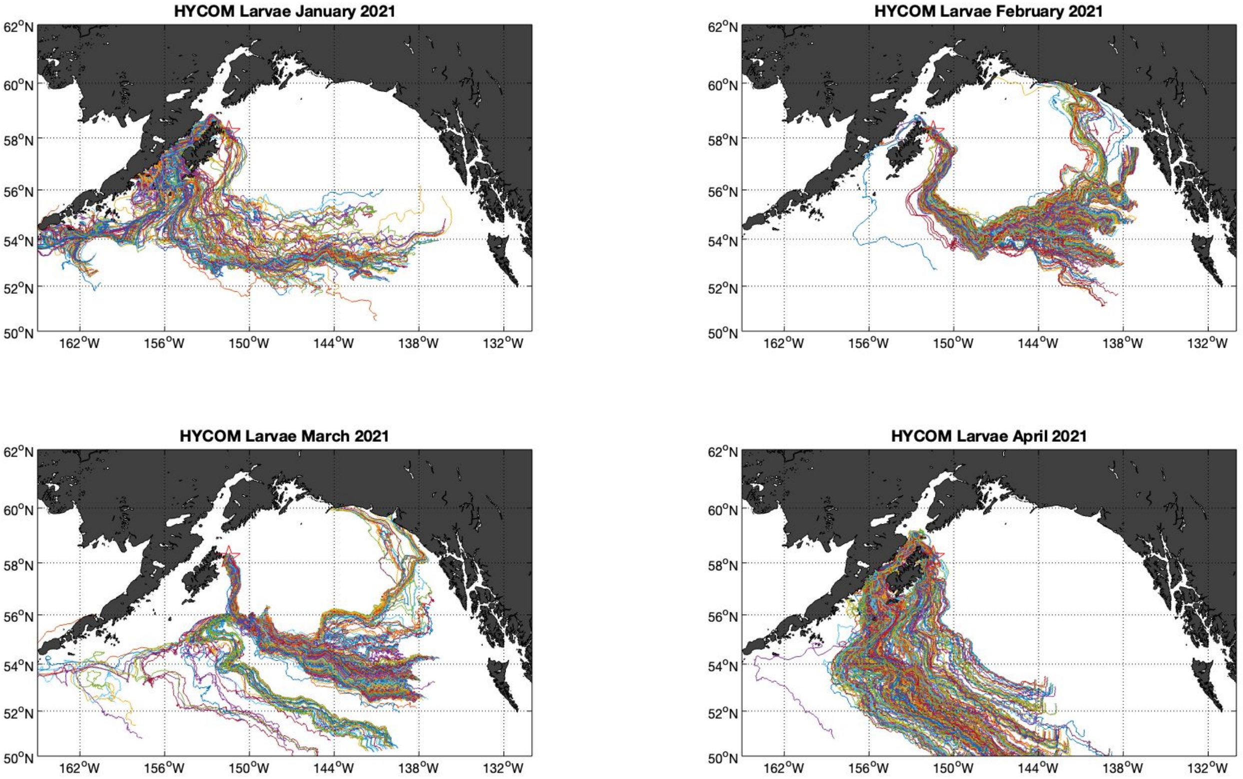

Supplementary Figure 9. HYCOM model output for a 200-day simulation of particles released off the northeast corner of Kodiak Island (58.3 N 151.5 W red star on each map) in 2021 on the 15th of A) January, B) February, C) March, and D) April. Each individual line represents an individual particle simulation.

Sample Site	Aleutians	eGOA	NBS	wGOA_EBS
1. Adak	99.2 (94)	0.3 (0)	0.3 (0)	0.3 (0)
2. West Attu	98.4 (47)	0.5 (0)	0.5 (0)	0.6 (0)
3. St. Lawrence	1 (0)	0.9 (0)	77.8 (22)	20.3 (5)
4. South Central NBS	2.2 (0)	1.9 (0)	32.4 (4)	63.5 (8)
5. West of Nome	0.4 (0)	0.4 (0)	26.4 (15)	72.8 (43)
6. East of St. Lawrence	1 (0)	1 (0)	12.9 (3)	85.1 (21)
7. Norton Sound	0.8 (0)	0.8 (0)	11 (4)	87.4 (26)
8. Eastern Bering Sea	9.1 (2)	1.1 (0)	1 (0)	88.8 (23)
9. Western GOA	0.9 (0)	1 (0)	1 (0)	97.1 (24)
10. Kodiak	1.1 (0)	1.2 (0)	1.1 (0)	96.6 (21)
11. Outside Cook Inlet	0.9 (0)	9.6 (2)	5.3 (1)	84.2 (20)
12. Central GOA	0.5 (0)	7.7 (4)	0.5 (0)	91.3 (50)
13. Eastern GOA	1.1 (0)	84.9 (22)	1 (0)	13 (3)

Supplementary Table 1. Mixture proportions and individual assignment for each summer adult sampling location with mixtures given in percent to each reporting group based on the inferred mixing proportions and with the number of individuals assigning to each reporting group in parentheses.

Sample Site	Aleutians	eGOA	NBS	wGOA_EBS
1. Near Sand Point 2021	0.4 (0)	1.9 (1)	0.4 (0)	97.4 (68)
2. Mitrofania Bay 2021	1 (0)	1.2 (0)	1.1 (0)	96.7 (22)
3. Sutwik Island 2021	0.6 (0)	2.5 (1)	0.5 (0)	96.5 (46)
4. South of Kodiak 2021	3.3 (1)	6.1 (2)	0.7 (0)	89.9 (31)
5. Shelikov Strait 2021	0.5 (0)	14.9 (7)	0.5 (0)	84 (40)
6. Shelikov Strait 2023	0.5 (0)	15.2 (8)	0.5 (0)	83.8 (47)
7. North Kodiak 2021	0.4 (0)	5.5 (3)	0.4 (0)	93.6 (53)
8. Cook Inlet outside 2021	0.2 (0)	9.8 (12)	0.2 (0)	89.8 (111)
9. PWS outside 2021	0.5 (0)	2.6 (1)	0.5 (0)	96.5 (48)
10. eGOA outside 2023	0.3 (0)	99.2 (97)	0.3 (0)	0.3 (0)
11. eGOA outside 2021	0.9 (0)	11.1 (3)	4.3 (1)	83.7 (24)
12. eGOA inside 2023	1.4 (0)	95.8 (16)	1.4 (0)	1.4 (1)

Supplementary Table 2. Mixture proportions and individual assignment for each juvenile sampling location with mixtures given in percent to each reporting group based on the inferred mixing proportions and the number of individuals assigning to each reporting group in parentheses.
